# Targeting the TIGIT/CD155-induced metabolic checkpoint in NK cells restores anti-tumor immunity and suppresses hepatocellular carcinoma growth

**DOI:** 10.3389/fimmu.2026.1790174

**Published:** 2026-05-01

**Authors:** Jiaojie Shu, Wei Yu, Yaping Shen, Xiaolin Liu

**Affiliations:** 1 Department of Breast Surgery, Zhoushan Hospital of Zhejiang Province, Zhoushan, China; 2 Department of Hepatobiliary and Pancreatic Surgery, The Second Affiliated Hospital of Jiaxing University, Jiaxing, China

**Keywords:** glycolysis, hepatocellular carcinoma, immunometabolism, natural killer cells, TIGIT/CD155

## Abstract

**Background:**

The TIGIT/CD155 axis is a key immune checkpoint in hepatocellular carcinoma (HCC), but its role in regulating natural killer (NK) cell metabolism and function remains unclear. This study investigates how this axis impairs NK cell anti-tumor immunity via metabolic reprogramming.

**Methods:**

An HCC mouse model was used for single-cell RNA sequencing (scRNA-seq) and bulk RNA sequencing (RNA-seq) to identify dysregulated pathways in TIGIT^high^ NK cells. A co-culture system consisting of CD155-overexpressing tumor cells and NK cells was established. Molecular interactions were examined by co-immunoprecipitation, western blotting, and immunofluorescence. NK-cell glycolytic activity was assessed by extracellular acidification rate, glucose uptake, and lactate production, and NK-cell function was evaluated by cytokine secretion and cytotoxicity assays. The involvement of the SHP-2/STAT3/GLUT1 axis was further examined using genetic and pharmacological interventions *in vitro* and in a xenograft model.

**Results:**

CD155 expression was increased in HCC tissues. TIGIT^high^ NK cells showed transcriptional features consistent with impaired glycolytic activity and functional suppression. CD155 engagement was associated with increased SHP-2 recruitment, reduced STAT3 phosphorylation, and lower GLUT1 expression, accompanied by decreased glycolytic activity. TIGIT blockade restored GLUT1 expression, glycolytic flux, and NK-cell effector function, including IFN-γ production, granzyme B expression, and cytotoxic activity. These effects were weakened by STAT3 inhibition or GLUT1 knockdown. *In vivo*, TIGIT blockade reduced tumor growth and was associated with increased metabolic and functional markers in NK cells, whereas STAT3 inhibition partially attenuated these effects.

**Conclusion:**

The TIGIT/CD155 axis is associated with NK-cell dysfunction in HCC through SHP-2-dependent suppression of STAT3/GLUT1-related glycolytic metabolism. These findings suggest that metabolic restoration may represent a potential strategy to improve NK-cell-mediated antitumor immunity in HCC.

## Graphical Abstract

**Figure:**
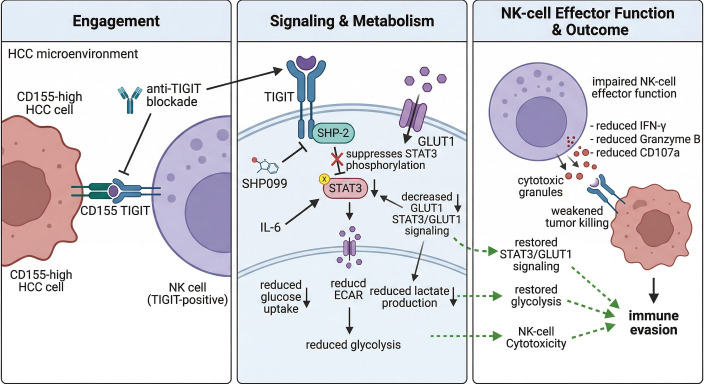
TIGIT–CD155 interaction recruits SHP-2 to inhibit the STAT3–GLUT1 axis, reprogram glycolytic metabolism, reduce NK cell cytotoxicity, and promote immune escape in HCC.

**Highlights**

- This study reveals a previously underappreciated mechanism by which TIGIT modulates the STAT3/GLUT1 signaling axis through SHP-2, thereby impairing NK cell function.
- This study reveals that high expression of CD155 in tumor cells significantly promotes upregulation of TIGIT in NK cells.
- This study provides evidence thats that TIGIT facilitates the formation of a complex between SHP-2 and STAT3, thereby inhibiting STAT3 phosphorylation.
- This study shows that elevated TIGIT expression is associated with reduced glycolysis and decreased GLUT1 expression in NK cells.
- This study identifies potential intervention targets within the TIGIT/SHP-2 pathway for immunotherapy in HCC.

## Introduction

Hepatocellular carcinoma (HCC) is the most common primary liver malignancy and remains a leading cause of cancer-related death worldwide. Its incidence has continued to increase in recent years, particularly in regions of Asia and Africa where hepatitis B virus or hepatitis C virus infection is prevalent (1, 2). Although immune checkpoint inhibitors (ICIs) have shown clinical benefit in a subset of patients with HCC, overall response rates remain limited and many patients still experience early disease progression and unsatisfactory long-term survival. These observations suggest that complex immune evasion mechanisms operate within the HCC tumor microenvironment (TME) (3, 4). A better understanding of the mechanisms underlying immune cell dysfunction in HCC may help improve the efficacy of immunotherapy (5, 6).

Natural killer (NK) cells are key effector cells of the innate immune system and contribute substantially to tumor immune surveillance through antigen-independent cytotoxicity (7, 8). In early-stage HCC, NK-cell activity has been associated with patient prognosis (9, 10). However, accumulating evidence indicates that intratumoral NK cells in HCC commonly display a functionally impaired state, characterized by reduced expression of cytotoxic molecules, diminished degranulation capacity, and low metabolic activity, all of which may weaken their antitumor effects (5, 8). Defining the regulatory pathways that contribute to NK-cell dysfunction, together with their upstream signals and metabolic basis, may provide useful insight for HCC immunotherapy (7, 11). NK-cell activation and maturation are closely linked to metabolic reprogramming. Previous studies have shown that the mTOR pathway is a central regulator of metabolic adaptation in NK cells, and that IL-15 promotes NK-cell development and activation partly through mTOR-dependent metabolic programming (12). Activated NK cells increase glucose uptake and glycolytic activity to support granule release, cytokine production, and immune synapse formation, indicating that glycolysis is important for maintaining cytotoxic function (13). In the TME, however, metabolic stress can impair NK-cell activity. For example, aberrant FBP1 expression suppresses glycolysis and is associated with defective NK-cell function during lung cancer progression (14). NK cells in tumors are also exposed to nutrient limitation, including glucose deprivation, and their metabolic status appears closely related to antitumor activity (15). Nevertheless, whether inhibitory receptors directly participate in NK-cell metabolic reprogramming remains incompletely understood, particularly with respect to the influence of TIGIT signaling on glycolysis.

Within the HCC immune microenvironment, NK-cell suppression has been associated with altered receptor expression patterns (6, 9). Among these receptors, T cell immunoreceptor with Ig and ITIM domains (TIGIT) has emerged as an important inhibitory checkpoint on NK cells. TIGIT is highly expressed in several solid tumors, including HCC, lung cancer, and breast cancer, and has been linked to NK-cell functional exhaustion (16). Its ligand, cluster of differentiation 155 (CD155; also known as PVR), is mainly expressed by tumor cells and can trigger inhibitory signaling upon binding to TIGIT (17, 18). Previous studies suggest that TIGIT may recruit Src homology 2 domain-containing phosphatase 2 (SHP-2) through its intracellular ITIM and ITSM motifs, thereby interfering with downstream pathways such as JAK/STAT signaling and modulating cellular function (19). However, current studies of TIGIT in NK cells have focused mainly on cytokine production and cytotoxic inhibition, whereas its possible role in metabolic regulation, especially glycolysis, remains insufficiently defined (20, 21). Given the central importance of glucose metabolism in NK-cell activation and cytotoxicity, whether TIGIT contributes to immune evasion by reshaping metabolic networks remains an important unanswered question (22, 23).

Glycolysis is a major metabolic pathway supporting the rapid energy demands of NK cells during effector responses (22, 24). Activated NK cells require enhanced glycolytic flux to sustain granzyme release, cytokine secretion, and proliferation (25). In this context, STAT3 and the downstream metabolic regulator GLUT1 are likely to play important roles (26). STAT3 phosphorylation has been reported to promote GLUT1 expression, thereby facilitating glucose uptake and metabolic activity that support NK-cell function (26). Evidence suggests that SHP-2 acts as a negative regulator by dephosphorylating STAT3, influencing its metabolic functions (19). However, direct evidence for a complete TIGIT/CD155–SHP-2–STAT3–GLUT1 signaling axis that links checkpoint activation to metabolic suppression of NK cells in the HCC microenvironment remains limited (23). In particular, systematic studies integrating multi-omics analyses with *in vivo* and *in vitro* functional validation are still lacking. Clarifying this regulatory network may improve understanding of NK-cell dysfunction in HCC (27).

Recent advances in single-cell multi-omics have enabled a more refined characterization of immune cell states, receptor interactions, and metabolic programs at the subpopulation level (28, 29). In the tumor immune microenvironment, single-cell RNA sequencing (scRNA-seq), combined with bulk RNA sequencing and ligand–receptor inference tools such as CellChat and CellPhoneDB, provides a useful framework for dissecting intercellular communication, signaling pathways, and metabolic features (30, 31). Integration of these approaches with *in vivo* and *in vitro* evidence may help define immune checkpoint-associated metabolic networks and provide support for mechanism-based therapeutic intervention (25).

In the present study, we aimed to characterize the metabolic features of TIGIT^+^ NK cells and explore their potential contribution to immune evasion in HCC, with particular attention to the possible involvement of the SHP-2/STAT3/GLUT1 axis in TIGIT–CD155-associated metabolic suppression. By examining the link between immune checkpoint signaling and cellular metabolic regulation, this work may provide additional insight into the molecular basis of NK-cell dysfunction in HCC and offer a rationale for strategies designed to restore antitumor immunity through metabolic modulation.

## Materials and methods

### scRNA-seq

To investigate the heterogeneity of TIGIT-associated regulation of NK-cell function, a subcutaneous Hepa1–6 HCC model was established in C57BL/6J mice. Normal liver tissues and tumor tissues were collected, mechanically dissociated, and digested with 0.2% collagenase IV (Sigma) and 0.01% DNase I (Roche) to generate single-cell suspensions enriched for tumor-infiltrating CD45^+^ immune cells. Samples from one mouse per group were pooled and immediately processed for single-cell capture and library preparation using the 10x Genomics Chromium Next GEM Single Cell 3′ Reagent Kits v3.1. Sequencing was performed on an Illumina NovaSeq 6000 platform at a minimum depth of 50,000 reads per cell. Raw data were processed using Cell Ranger (v7.1.0) for alignment to the mouse reference genome (mm10) and UMI counting, yielding the final gene expression matrix.

All downstream analyses were performed in R (v4.2.1). Data normalization, quality control, and integration were conducted using Seurat (v4.3.0). Cells with >25% mitochondrial gene expression or with <200 or >5000 detected genes were excluded. Sample integration was performed using Harmony (v1.1.0). SCTransform normalization was applied, followed by principal component analysis (PCA) based on the top 30 principal components. Uniform manifold approximation and projection (UMAP) was used for dimensionality reduction, and clustering was performed with the Louvain algorithm at a resolution of 0.8. Cell-type annotation was based on the CellMarker database (http://bio-bigdata.hrbmu.edu.cn/CellMarker/) combined with published literature. NK cells were extracted according to canonical markers (Nkg7, Klrd1, and Gzmb) for further subset analysis. Cells were stratified into TIGIT^+^ and TIGIT^-^ groups according to the median TIGIT expression level. Differentially expressed genes (DEGs) between groups were identified using the Wilcoxon rank-sum test (log_2_FC ≥ 0.25, *p* < 0.05), followed by Benjamini–Hochberg correction. Downregulated DEGs were further subjected to KEGG pathway enrichment analysis using the clusterProfiler package (v4.8.1) with the mmu database (pAdjustMethod = BH, adjusted *p* < 0.05). Chord diagrams were generated with circlize (v0.4.15) and GOplot (v1.0.2). This analysis was primarily used to characterize immune-cell subsets and identify candidate pathways.

### Bulk RNA-seq

Total RNA was simultaneously extracted from tumor tissues (n = 4) and matched normal liver tissues (n = 4) obtained from the same mouse model. RNA was isolated using TRIzol reagent, and integrity was assessed with an Agilent Bioanalyzer 2100 (RIN > 8.0). Libraries were prepared using the NEBNext Ultra RNA Library Prep Kit and sequenced on an Illumina NovaSeq 6000 platform with 150 bp paired-end reads. Raw sequencing data were processed with Trimmomatic (v0.39) to remove low-quality reads and adaptor contamination, followed by alignment to the reference genome (mm10) using STAR (v2.7.10a). Gene quantification was performed with featureCounts, and differential expression analysis was conducted using DESeq2 (v1.38.3). Differentially expressed genes were defined as those with |log_2_FC| > 1 and *p* < 0.05. To further prioritize bulk DEGs, LASSO regression was applied using the glmnet package (v4.1-8). A normalized expression matrix was used as input, with tumor versus normal status as the response variable, and the penalty parameter λ was determined by 10-fold cross-validation. Selected feature genes were then imported into GeneMANIA for interaction network construction and connectivity analysis.

### Construction of mouse models and animal ethics

Male C57BL/6J mice aged 6–8 weeks and weighing 18–22 g were housed under specific pathogen-free conditions with controlled temperature and humidity under a 12-h light/dark cycle, with free access to food and water. Mice were randomly assigned to groups before the experiments. To generate a subcutaneous HCC model, Hepa1–6 cells stably overexpressing CD155 (Hepa1-6-CD155) were established by lentiviral transduction and puromycin selection. Cells in the logarithmic growth phase were harvested, resuspended in PBS at 5 × 10^6^ cells/100 μL, and injected subcutaneously into the right axilla. Tumors became detectable within approximately 7–10 days. Tumor volume was monitored regularly using the formula: volume = 1/2 × length × width^2^. When the mean tumor volume reached approximately 100 mm³, mice received NK-cell treatment. Three groups were included: NK cells pretreated with isotype-control IgG, NK cells pretreated with anti-TIGIT antibody, and NK cells pretreated with anti-TIGIT antibody plus the STAT3 inhibitor JSI-124. NK cells were administered by tail vein injection at 2 × 10^6^ cells per mouse. Each group included 15 mice. Treatment was given once every 3 days for a total of 2–3 administrations. Cell viability was confirmed by trypan blue exclusion before injection, and only preparations with >95% viability were used.

Mice were sacrificed on day 28 after the final administration. Tumor tissues, liver, spleen, and additional organs were collected. Final tumor weight and volume were recorded, and tumor growth inhibition was calculated. Tumors were paraffin-embedded and sectioned for hematoxylin and eosin (H&E) staining, Ki67 immunohistochemistry (IHC), and TUNEL staining. Tumor-infiltrating CD45^+^NKp46^+^ NK cells were isolated by fluorescence-activated cell sorting (FACS) for western blotting (WB) and reverse transcription quantitative PCR (RT-qPCR) analyses of SHP-2, p-STAT3, GLUT1, and glycolysis-related enzymes, including HK2 and LDHA. ImageJ was used for quantitative analysis of IHC and immunofluorescence images, including NK-cell infiltration and functional markers such as IFN-γ and granzyme B. All animal experiments were approved by the institutional ethics committee of The Second Affiliated Hospital of Jiaxing University and were performed in accordance with national guidelines for laboratory animal care. Experimental procedures were independently conducted by two investigators for cross-validation.

### Isolation and processing of primary NK cells

Primary NK cells were isolated from the spleen and liver of C57BL/6J mice. Tissues were excised under sterile conditions, rinsed in pre-chilled RPMI-1640 medium, and mechanically dissociated. The suspensions were digested with collagenase IV (1 mg/mL, Sigma) and DNase I (50 U/mL, Roche) in a 37 °C shaking water bath for 30 min. After filtration through a 70-μm cell strainer, cells were washed and resuspended in PBS. CD3^-^NK1.1^+^ NK cells were then purified by negative selection using the NK Cell Isolation Kit (Miltenyi Biotec). Purity was verified by flow cytometry using FITC-conjugated anti-CD3 and APC-conjugated anti-NK1.1 antibodies on a BD FACSCanto II cytometer, and preparations with >90% purity were used. Viability was assessed by trypan blue exclusion, and freshly isolated cells were used within 24 h.

For genetic manipulation, TIGIT and SHP-2 overexpression constructs were generated using the GV492 vector backbone (GeneChem). For GLUT1 knockdown, shRNA sequences targeting GLUT1 (sh-GLUT1#1 and sh-GLUT1#2) were cloned into lentiviral vectors. NK cells were infected at a multiplicity of infection of 50 in the presence of Polybrene (8 μg/mL). Spin infection was performed in six-well plates at 37 °C and 1200 rpm for 90 min, followed by replacement with fresh complete medium containing 10% fetal bovine serum (FBS) and IL-2 (200 IU/mL). Cells were collected 48–72 h later, and stable transfectants were selected using puromycin (2 μg/mL). Transduction efficiency was assessed by RT-qPCR and confirmed by WB. Before functional assays, all transduced NK cells were maintained in IL-2 (200 IU/mL) for at least 24 h.

### Anti-TIGIT blocking antibody treatment and rescue experiments in the co-culture system

To evaluate the role of CD155–TIGIT signaling in NK-cell glycolytic regulation, rescue experiments were performed using a co-culture system consisting of CD155-overexpressing Hepa1–6 cells and NK cells. A blocking anti-mouse TIGIT antibody (clone 1G9, Cat# BE0274, BioXcell, West Lebanon, USA) or isotype-control IgG (mouse IgG1, clone MOPC-21, Cat# BE0083, BioXcell) was used. Primary NK cells were co-cultured with CD155-overexpressing Hepa1–6 cells at an effector-to-target ratio of 10:1 in high-glucose DMEM complete medium (10% FBS, 1% penicillin–streptomycin, IL-2–200 IU/mL) with a final antibody concentration of 5 μg/mL. The experimental groups were as follows: Control (NK cells alone), CD155 (CD155-overexpressing tumor cells + NK cells), CD155+IgG (CD155-overexpressing tumor cells + NK cells + isotype-control IgG), and CD155+anti-TIGIT (CD155-overexpressing tumor cells + NK cells + anti-TIGIT blocking antibody). After 48 h of co-culture, cells were collected for subsequent metabolic analyses, including ECAR measurement on the Seahorse XF96 platform, glucose uptake using the 2-NBDG fluorescent probe, lactate concentration in the supernatant, and RT-qPCR analysis of glycolysis-related enzyme mRNA levels (32).

### Cultivation and treatment of tumor cell lines

The murine HCC cell line Hepa1-6 (ATCC^®^ CRL-1830™) was maintained in high-glucose DMEM supplemented with 10% FBS (Gibco) and 1% penicillin–streptomycin (Gibco) at 37 °C in a humidified incubator containing 5% CO_2_. Cells were passaged using 0.25% trypsin-EDTA (Gibco) and maintained at 70%–80% confluence. Cells from passages 3–10 were used.

Stable CD155 overexpression was achieved using the GV492 lentiviral system (GeneChem). The overexpression construct contained full-length CD155 cDNA together with an EGFP reporter. Lentiviral particles were produced in 293T cells by co-transfection with three plasmids. Supernatants were collected 48 h later and concentrated by ultrafiltration to achieve titers >1 × 10^8^ TU/mL. Hepa1–6 cells were infected at an MOI of 20 in the presence of Polybrene (8 μg/mL), followed by spin infection at 37 °C and 1200 rpm for 90 min. Fresh medium was added after infection, and puromycin selection (2 μg/mL) was initiated 72 h later for 7 days. Stable clones were expanded for subsequent co-culture and animal experiments. All procedures were conducted under BSL-2 conditions and approved by the institutional biosafety committee.

### Western blot

Protein expression of TIGIT, CD155, SHP-2, STAT3, phosphorylated STAT3 (p-STAT3), and GLUT1 was examined by WB. Total protein was extracted using RIPA buffer (P0013B, Beyotime, China) and quantified with a BCA assay kit (23225, Thermo Fisher Scientific, USA). Equal amounts of protein (20 μg) were separated by 10% SDS–PAGE and transferred onto PVDF membranes (IPVH00010, Millipore, USA). Membranes were blocked with 5% non-fat milk for 1 h at room temperature and incubated overnight at 4 °C with the following primary antibodies: rabbit anti-TIGIT (20574, Cell Signaling Technology, USA, 1:1000), rabbit anti-CD155 (ab314064, Abcam, UK, 1:1000), rabbit anti-SHP-2 (3397, Cell Signaling Technology, USA, 1:1000), rabbit anti-STAT3 (4904, Cell Signaling Technology, USA, 1:1000), rabbit anti-p-STAT3 (Tyr705) (9145, Cell Signaling Technology, USA, 1:1000), mouse anti-GLUT1 (ab238050, Abcam, UK, 1:1000), and rabbit anti-β-actin (4970, Cell Signaling Technology, USA, 1:5000). After primary antibody incubation, the membranes were incubated at room temperature for 1 hour with HRP-conjugated goat anti-rabbit IgG (7074S, Cell Signaling Technology, USA, 1:5000) or goat anti-mouse IgG (sc-2005, Santa Cruz Biotechnology, USA, 1:5000) as secondary antibodies. Protein signals were developed using ECL chemiluminescent substrate (32106, Thermo Fisher Scientific, USA) and analyzed with ImageJ software (v1.53t; NIH, USA).

### RT-qPCR

Total RNA was extracted from tissues using the Trizol reagent (15596026, Invitrogen, Thermo Fisher Scientific). The concentration and purity of the extracted RNA were assessed with a Nanodrop 2000 spectrophotometer (1011U, Nanodrop, Thermo Fisher Scientific USA). cDNA synthesis was performed using the PrimeScript RT reagent Kit (RR047A, Takara, Japan). Quantitative PCR was carried out using Fast SYBR Green PCR Master Mix (RR820A, Takara, Japan) on an ABI PRISM 7300 system. The cycling conditions included initial denaturation at 95 °C for 5 min, followed by 40 cycles of 95 °C for 30 s, 57 °C for 30 s, and 72 °C for 30 s. All samples were analyzed in triplicate. β-actin served as the internal reference, and relative gene expression was calculated using the 2^-ΔΔCt^ method. Primer sequences are provided in **Supplementary Table S1**.

### Co-immunoprecipitation

The Co-IP assay was performed to examine the interaction between SHP-2 and STAT3. Total protein was extracted from NK cells using RIPA buffer, and at least 500 μg protein per sample was used. Lysates were pre-cleared with isotype IgG and Protein A/G magnetic beads (Thermo Scientific, Cat# 88802) for 1 h at 4 °C. Samples were then incubated overnight at 4 °C with 1–2 μg anti-SHP-2 or anti-STAT3 antibody. Protein A/G magnetic beads were added for an additional 4 h, followed by five washes with IP buffer. Bound complexes were eluted by boiling in SDS loading buffer and subjected to SDS–PAGE and WB. IgG immunoprecipitation served as the negative control. All assays were repeated three times.

### Functional analysis of glycolysis

Glycolytic function was evaluated by measuring extracellular acidification rate (ECAR), glucose uptake capacity, and lactate production. ECAR was measured using the Seahorse XF96 Extracellular Flux Analyzer (Agilent Technologies, USA) with the Glycolysis Stress Test Kit (Cat# 103020-100). NK cells were seeded in poly-L-lysine-coated Seahorse 96-well plates at 1 × 10^5^ cells per well and equilibrated in glucose-free assay buffer for 1 h. Glucose (10 mM), oligomycin (1 μM), and 2-deoxy-D-glucose (2-DG; 50 mM) were sequentially injected. Basal glycolysis and glycolytic reserve were calculated using Seahorse Wave software. Five technical replicates were included for each condition.

Glucose uptake was assessed using 2-NBDG (Thermo Fisher Scientific, Cat# N13195) at a final concentration of 20 μM. NK cells were incubated in serum-free medium for 30 min, followed by incubation with 2-NBDG for an additional 30 min. Fluorescence intensity was measured in the FITC channel on a BD Fortessa flow cytometer, and geometric mean fluorescence intensity was used for quantification.

Lactate levels in culture supernatants were determined using a Lactate Assay Kit (BioVision, Cat# K607-100). Absorbance was measured at 570 nm, and lactate concentrations were calculated from a standard curve. At least three biological replicates were included.

### Flow cytometry

NK-cell function was evaluated by multiparameter flow cytometry. After treatment, NK cells were washed twice with PBS and adjusted to 1 × 10^6^ cells/mL. Cells were stained with antibodies against CD107a, IFN-γ, and granzyme B. For CD107a degranulation assays, antibody and GolgiStop were added during a 4-h incubation at 37 °C. For intracellular cytokine staining, cells were fixed and permeabilized using BD Cytofix/Cytoperm buffer. Fluorescence minus one controls were included to define gating boundaries. Data acquisition was performed on a BD FACSVerse system and analyzed using FlowJo v10.

### ELISA

Cytokine secretion was measured using mouse IFN-γ ELISA and TNF-α ELISA kits (eBioscience). Supernatants from treated co-culture systems were collected after centrifugation and analyzed within 48 h. Samples and standards were assayed in triplicate, and absorbance was measured at 450 nm. Cytokine concentrations were calculated from standard curves.

### Tumor killing assay (carboxyfluorescein succinimidyl ester/PI labeling and lactate dehydrogenase release)

NK-cell cytotoxicity against Hepa1–6 cells was evaluated using CFSE/PI staining and LDH release assays. For CFSE/PI analysis, Hepa1–6 target cells were labeled with 5 μM CFSE at 37 °C for 10 min, washed, and co-cultured with treated NK cells at an effector-to-target ratio of 5:1 for 4–6 h. Propidium iodide (PI, 1 μg/mL) was then added, and CFSE^+^PI^+^ cells were quantified by flow cytometry. Target cells cultured alone served as the spontaneous death control.

To further quantify NK cell-mediated tumor cell lysis, LDH release in the culture supernatant was measured using the Pierce™ LDH Cytotoxicity Assay Kit (Thermo Fisher Scientific, USA). After co-culture, 400 μL of supernatant from each group was collected and centrifuged at 10,000 × g for 5 min to remove cellular debris. The clarified supernatants were then subjected to LDH measurement. Absorbance was recorded at 490 and 680 nm after termination of the reaction. Cytotoxicity was calculated as follows: cytotoxicity (%) = [(experimental release − spontaneous release)/(maximum release − spontaneous release)] × 100%.

### Histological, immunohistochemical, and TUNEL staining

Tumor tissues were collected immediately after euthanasia, fixed in 4% paraformaldehyde for 24 h, embedded in paraffin, and sectioned at 4 μm. H&E staining was performed to assess overall tissue architecture and necrotic areas.

Apoptosis was evaluated using the Roche *In Situ* Cell Death Detection Kit (POD). TUNEL-positive cells were identified by brown-yellow nuclear staining. Positive cells were counted in five randomly selected high-power fields, and the TUNEL-positive rate was calculated.

Cell proliferation was assessed by immunohistochemical staining for Ki67. After deparaffinization and rehydration, sections underwent antigen retrieval in citrate buffer. Endogenous peroxidase activity was blocked with 3% hydrogen peroxide, followed by blocking of nonspecific binding. Sections were then incubated overnight at 4 °C with anti-Ki67 antibody (1:200, Abcam), followed by incubation with an HRP-conjugated secondary antibody and visualization using DAB. Hematoxylin was used for counterstaining.

To evaluate NK-cell infiltration, immunohistochemical staining was performed using anti-CD49b (1:200) and anti-NKp46 (1:200) antibodies. Images were acquired using a Leica DM4000B microscope. For each sample, at least five randomly selected high-power fields were analyzed using ImageJ to quantify the positive area or positive cell number. Uniform background correction and threshold settings were applied to all images.

### Sorting of NK cells via FACS

To examine the effects of the TIGIT–CD155 axis on NK-cell metabolic status, tumor-infiltrating NK cells were isolated from mouse tumor tissues. Fresh tumor tissues were rinsed with PBS and digested at 37 °C for 30–40 min in a solution containing collagenase IV (1 mg/mL, Sigma) and DNase I (50 U/mL, Roche), with gentle pipetting performed intermittently to facilitate dissociation. The resulting cell suspension was passed through a 70-μm cell strainer, washed with PBS, and treated with red blood cell lysis buffer. Cells were then stained with fluorescently labeled antibodies against CD45 and NKp46. CD45^+^NKp46^+^ cells were sorted using a FACSAria II cell sorter (BD Biosciences). Each tumor sample was processed individually. Sorted NK cells were immediately used for protein and RNA extraction.

### Statistical analysis methods

Statistical analyses were performed using GraphPad Prism 10.0 and R (v4.2). Data normality was assessed using the Shapiro–Wilk test. For normally distributed data, comparisons were performed using Student’s t-test or one-way analysis of variance (ANOVA). For non-normally distributed data, the Mann–Whitney U test or Kruskal–Wallis test was used. Tumor volume changes over time were analyzed using two-way ANOVA. Survival was evaluated by Kaplan–Meier analysis with the log-rank test. Correlations were assessed using Pearson or Spearman correlation analysis as appropriate. All tests were two-sided, and *P* < 0.05 was considered statistically significant.

## Results

### Identification of TIGIT downstream signaling pathways and CD155 targets via integrated scRNA-seq and bulk RNA-seq

**Figure 1:**
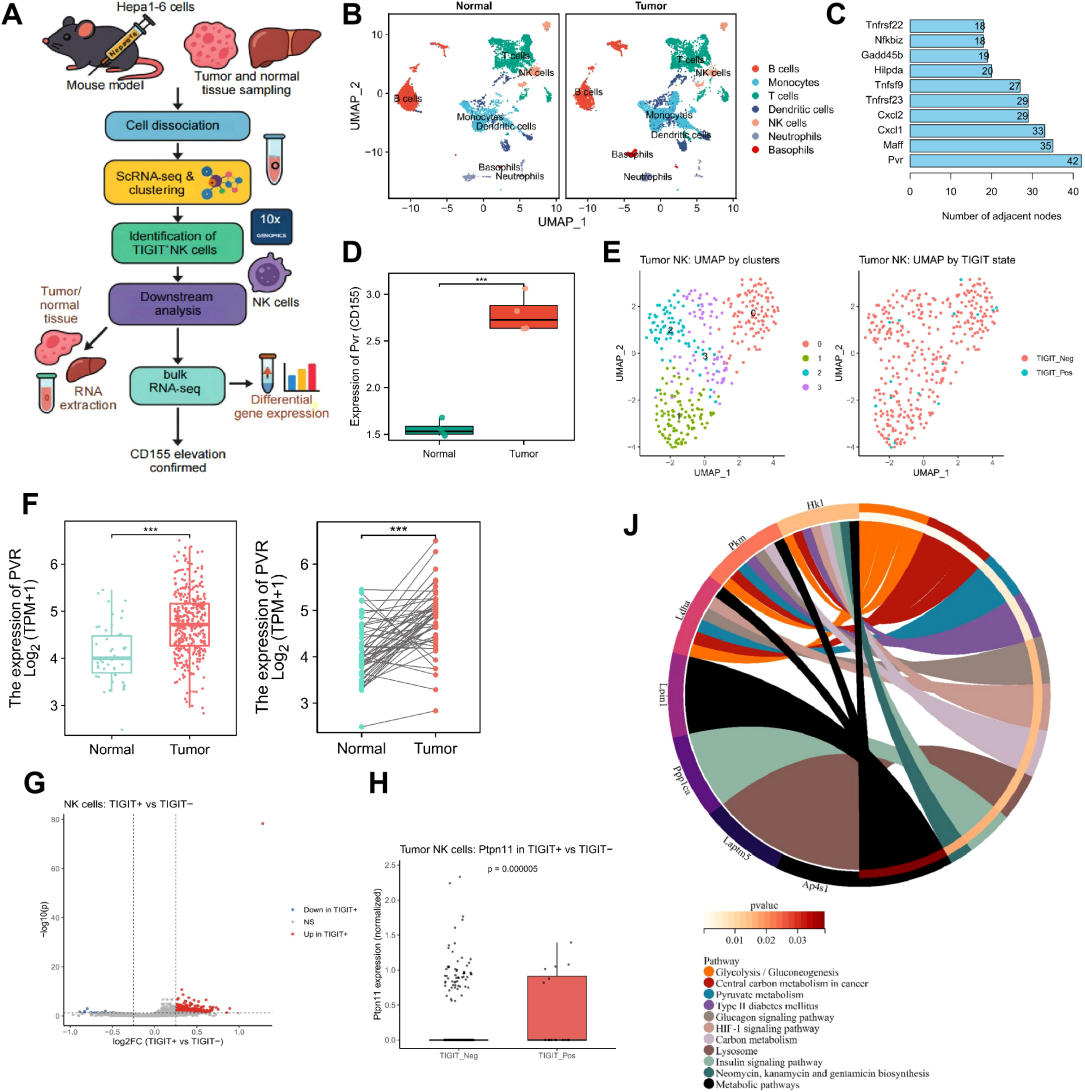
Identification of TIGIT^+^ NK cells subsets and their inhibitory pathways via integrated scRNA-seq and Bulk RNA-seq. **(A)** Workflow schematic of integrated scRNA-seq and bulk RNA-seq analyses; **(B)** UMAP visualization of immune cells from normal liver and hepatocellular carcinoma tissues identified by scRNA-seq; **(C)** Network topology analysis showing node connectivity of candidate key genes; **(D)** Bulk RNA-seq analysis of Pvr (CD155) expression in normal versus tumor tissues; **(E)** Expression of CD155 (PVR) in unpaired and paired HCC tissues and normal liver tissues from the TCGA database. For the unpaired analysis, Normal (n = 50) and Tumor (n = 374); for the paired analysis, n = 50. **(F)** UMAP visualization of tumor-infiltrating NK cells. Left: subclusters distinguished by clustering analysis, shown in distinct colors; Right: NK cells stratified by TIGIT expression, with TIGIT^−^ cells in red and TIGIT^+^ cells in blue; **(G)** Volcano plot of differentially expressed genes between TIGIT-high and TIGIT-low NK cells; **(H)** Expression levels of Ptpn11 in TIGIT^+^ versus TIGIT^−^ NK cell subsets; **(I)** KEGG pathway enrichment of downregulated genes, presented as a chord diagram. ***p < 0.001.

A subcutaneous HCC model was established in C57BL/6J mice by inoculation of Hepa1–6 cells. At the tumor progression stage, subcutaneous tumor tissues and normal liver tissues were collected. Immune cells were isolated and subjected to scRNA-seq using the 10x Genomics platform, enabling the annotation of multiple immune cell populations, including NK cells. NK cells were subsequently extracted for subset analysis. In parallel, bulk RNA-seq was performed on tumor tissues and normal liver tissues (**Figure 1A**). UMAP based on the top 30 principal components identified 25 distinct clusters (**Supplementary Figure S1A**). Cell-type annotation was performed by integrating well-established lineage markers from the literature with the CellMarker database, leading to the identification of seven major immune-cell populations in normal liver and tumor tissues, including B cells, monocytes, T cells, dendritic cells, NK cells, neutrophils, and basophils (**Figure 1B**, **Supplementary Figure S1B**). Comparative analysis revealed clear differences in cellular composition between tumor and normal liver tissues, with relatively lower proportions of B cells, T cells, and NK cells and relatively higher proportions of monocytes, dendritic cells, and granulocytes in tumor tissues (**Supplementary Figure S1C**). Given the highly specialized immune-tolerant nature of normal liver, this comparison was intended primarily to illustrate the overall immune contexture of tumor tissues rather than to directly infer absolute changes in immune-cell abundance.

To identify candidate molecular determinants, we next constructed feature models and interaction networks based on the differential expression matrix. Bulk transcriptomic analysis identified 266 DEGs (**Supplementary Figure S2A**). LASSO regression was then applied to prioritize disease-relevant DEGs, and six feature genes (Pvr, Cxcl1, Maff, Cd96, Las1l, and Tnfrsf23) were retained at the λ_min value (**Supplementary Figure S2B**). GeneMANIA-based interaction analysis further showed that Pvr (encoding CD155) had the highest connectivity in the interaction network, with 42 adjacent nodes, exceeding those of Maff (35), Cxcl1 (33), Tnfrsf23 (29), and Tnfsf9 (27) (**Figure 1C**). Moreover, CD155 expression was significantly higher in tumor tissues than in normal liver tissues in the mouse dataset (**Figure 1D**). Consistently, TCGA data showed that CD155 (PVR) expression was also higher in HCC tissues than in normal liver tissues (**Figure 1E**).

Because NK cells are major effector cells in tumor immune surveillance, we next examined TIGIT-related transcriptional features within the NK-cell compartment. Tumor-infiltrating NK cells were further divided into four clusters (0–3) and stratified into TIGIT^+^ and TIGIT^-^ subsets according to TIGIT expression (**Figure 1F**). Differential expression analysis between these two subsets identified 272 DEGs, including 82 downregulated genes and 190 upregulated genes (**Figure 1G**). Among the upregulated genes, Ptpn11, which encodes SHP-2, was significantly increased in TIGIT^+^ NK cells (**Figure 1H**). KEGG enrichment analysis of the downregulated genes, visualized with chord diagrams, revealed significant enrichment in glycolysis/gluconeogenesis, central carbon metabolism in cancer, the HIF-1 signaling pathway, and PI3K–Akt/insulin signaling axes (**Figure 1I**), collectively indicating that TIGIT^+^ NK cells exhibit restricted metabolic activity.

Together, the integrated single-cell and bulk transcriptomic analyses identified CD155 as a prominently upregulated molecule in HCC tissues and a candidate upstream ligand associated with TIGIT-related NK-cell dysfunction. The data further support an association between TIGIT expression, SHP-2-related signaling, and reduced glycolysis-associated transcriptional programs in tumor-infiltrating NK cells, providing a basis for subsequent mechanistic investigation.

### Overexpression of CD155 in tumor cells promotes TIGIT-mediated recruitment of SHP-2 and inhibits STAT3 signaling activity

**Figure 2:**
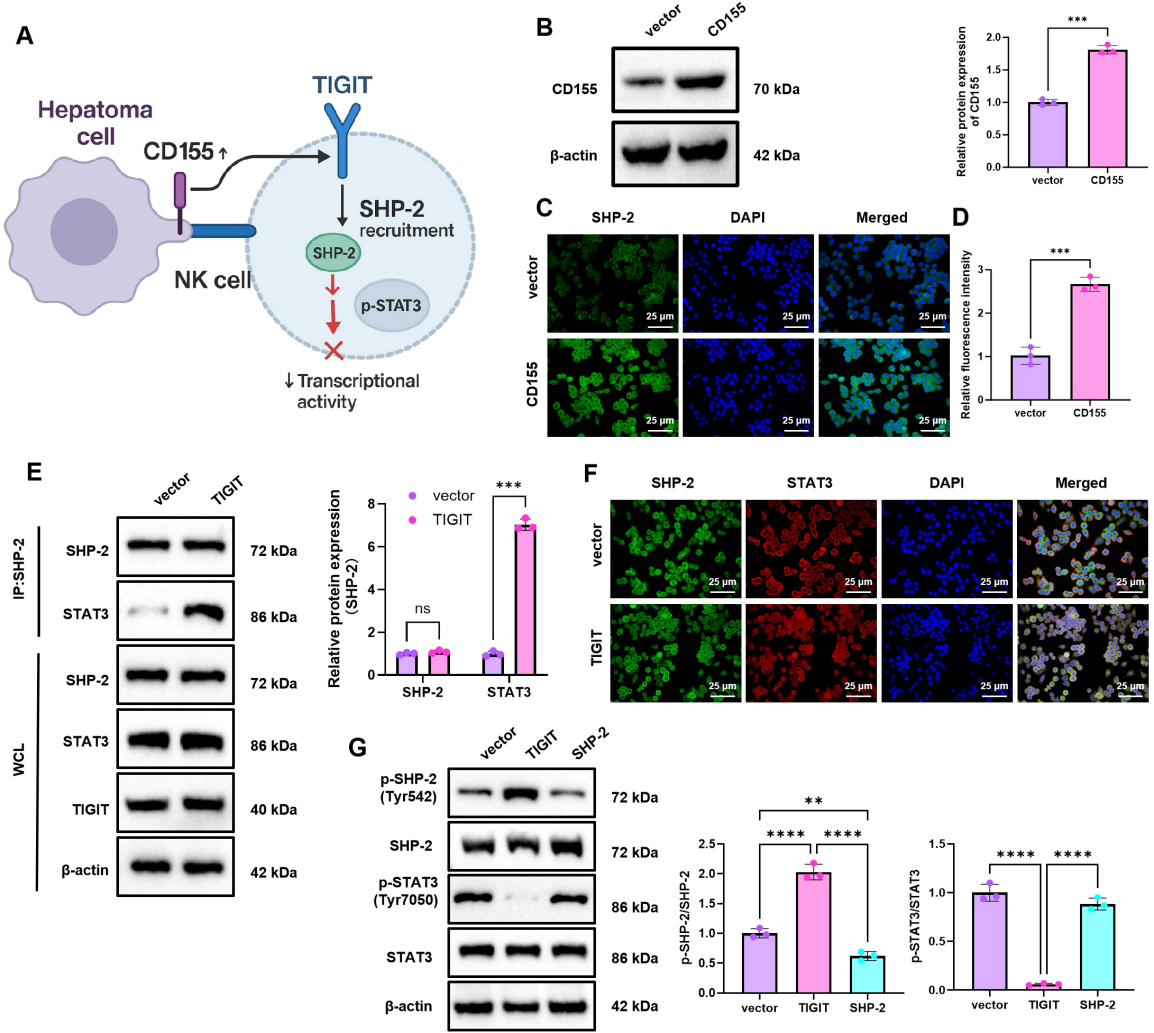
Validation of CD155-TIGIT signaling in SHP-2 recruitment and STAT3 pathway inhibition. **(A)** Schematic representation of the regulatory mechanism by which tumor cell CD155 modulates the SHP-2/STAT3 axis via TIGIT; **(B)** WB analysis of CD155 protein expression levels in Hepa1–6 cells; **(C)** Immunofluorescence staining showing SHP-2 expression and localization in co-cultured NK cells, scale bar: 25 µm; **(D)** Quantitative analysis of immunofluorescence signal intensity from panel C; **(E)** Co-IP assay demonstrating the interaction between SHP-2 and STAT3 in TIGIT-overexpressing NK cells; **(F)** Immunofluorescence colocalization analysis illustrating the distribution of SHP-2 and STAT3 in TIGIT-overexpressing NK cells, scale bar: 25 µm; **(G)** WB analysis of SHP-2, total STAT3, and its phosphorylated form p-STAT3 (Tyr705) protein levels in TIGIT or SHP-2-overexpressing NK cells. Experiments were conducted in triplicate. * indicates a statistically significant difference between groups, **p < 0.01, ***p < 0.001, ****p < 0.0001.

To examine whether tumor-derived CD155 modulates downstream signaling in NK cells through TIGIT, we established a Hepa1–6 murine HCC cell line with stable CD155 overexpression. These cells were co-cultured with primary NK cells isolated from normal mice to evaluate the effects of CD155–TIGIT interaction on SHP-2 expression and localization in NK cells, as well as on STAT3 signaling activity (**Figure 2A**). WB analysis confirmed a marked increase in CD155 protein expression in CD155-overexpressing Hepa1–6 cells relative to control cells (**Figure 2B**). Under co-culture conditions, immunofluorescence staining showed that SHP-2 expression in NK cells was higher in the CD155-overexpressing group than in the vector group (**Figures 2C, D**). To further assess whether TIGIT influences the interaction between SHP-2 and STAT3, a TIGIT-overexpressing NK-cell model was generated. Co-immunoprecipitation analysis showed enhanced SHP-2–STAT3 interaction in the TIGIT-overexpressing group compared with the vector group, suggesting that TIGIT expression is associated with increased recruitment of SHP-2 to STAT3-related signaling complexes (**Figure 2E**). Immunofluorescence co-localization experiments also confirmed increased co-localization signals of SHP-2 and STAT3 in the TIGIT overexpression group (**Figure 2F**).

To determine whether TIGIT-associated SHP-2 recruitment influences STAT3 signaling activity, TIGIT or SHP-2 was overexpressed in NK cells, and the expression and phosphorylation status of relevant proteins were examined. Compared with the vector group, TIGIT overexpression increased p-SHP-2 (Tyr542) and reduced p-STAT3 (Tyr705), whereas total STAT3 protein levels remained largely unchanged. By contrast, SHP-2 overexpression alone increased total SHP-2 abundance but did not produce a clear change in p-SHP-2 (Tyr542), p-STAT3 (Tyr705), or total STAT3 levels (**Figure 2G**). These results indicate that TIGIT promotes SHP-2 expression together with its phosphorylation-dependent activation, thereby leading to STAT3 dephosphorylation, whereas increased SHP-2 abundance alone is insufficient to trigger this effect.

In summary, these results support a model in which increased CD155 expression in tumor cells is associated with enhanced TIGIT-dependent SHP-2 recruitment in NK cells and reduced STAT3 phosphorylation activity.

### Overexpression of CD155 on tumor cells inhibits glycolytic activity in NK cells

**Figure 3:**
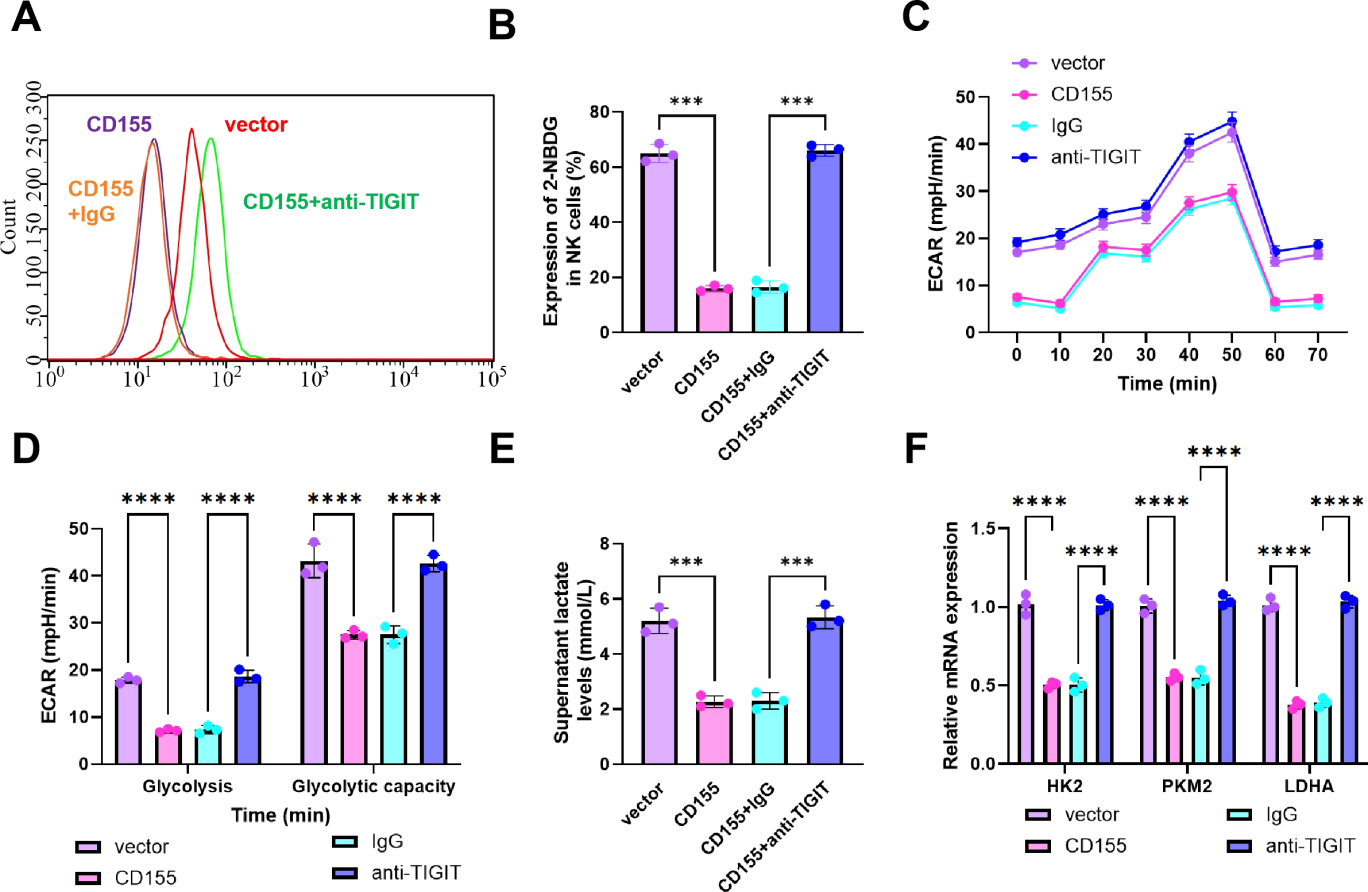
Tumor cells overexpressing CD155 suppress glycolytic capacity of co-cultured NK cells. **(A)** flow cytometry analysis of 2-NBDG fluorescence intensity in co-cultured NK cells; **(B)** Quantitative assessment of glucose uptake in NK cells; **(C)** Basal glycolytic flux (ECAR) of co-cultured NK cells measured using the Seahorse XF platform; **(D)** Quantitative analysis of glycolysis and glycolytic capacity; **(E)** Measurement of lactate concentration in cell supernatant using a lactate assay kit; **(F)** RT-qPCR analysis of mRNA expression levels of key glycolytic enzymes (HK2, PKM2, LDHA) in NK cells. Experiments were performed in triplicate. * indicates *p* < 0.05, ***p < 0.001, ****p < 0.0001 between groups.

To determine whether CD155-mediated TIGIT signaling influences glycolytic metabolism in NK cells, primary mouse NK cells were co-cultured with Hepa1–6 cells stably overexpressing CD155. Four groups were analyzed: Control, CD155, CD155+IgG, and CD155+anti-TIGIT. Glucose uptake was first assessed using the fluorescent glucose analog 2-NBDG. Compared with the Control group, 2-NBDG fluorescence intensity in NK cells was significantly reduced in both the CD155 and CD155+IgG groups, whereas no significant difference was observed between these two groups, indicating that CD155 overexpression was associated with reduced glucose uptake and that the isotype IgG control did not measurably affect this response. In contrast, 2-NBDG fluorescence intensity was significantly increased in the CD155+anti-TIGIT group relative to the CD155+IgG group, suggesting partial restoration of glucose uptake after TIGIT blockade (**Figures 3A, B**). Glycolytic flux was further assessed by Seahorse XF analysis of ECAR. Both basal glycolysis and glycolytic capacity were significantly decreased in the CD155 and CD155+IgG groups compared with the Control group, with no significant difference between the two groups. In contrast, both parameters were significantly increased in the CD155+anti-TIGIT group compared with the CD155+IgG group, indicating recovery of glycolytic function following TIGIT blockade (**Figures 3C, D**).

Consistent with these findings, lactate measurement in culture supernatants showed that lactate release from NK cells was markedly reduced in the CD155 and CD155+IgG groups relative to the Control group, again with no significant difference between the two groups. Lactate production was significantly increased in the CD155+anti-TIGIT group compared with the CD155+IgG group (**Figure 3E**). RT-qPCR analysis further showed that the mRNA expression levels of the glycolysis-related genes HK2, PKM2, and LDHA were significantly decreased in the CD155 and CD155+IgG groups relative to the Control group, whereas all three transcripts were significantly increased in the CD155+anti-TIGIT group compared with the CD155+IgG group (**Figure 3F**).

Together, these results suggest that CD155 overexpression in tumor cells is associated with suppression of glycolytic metabolism in NK cells, whereas TIGIT blockade partially reverses this effect. These findings support a role for the CD155–TIGIT axis in the regulation of NK-cell metabolic state and suggest that this pathway may influence NK-cell functional fitness by limiting glycolytic activity.

### Intervention in TIGIT/SHP-2/STAT3 pathway modulates GLUT1 expression and influences NK cell glycolysis

**Figure 4:**
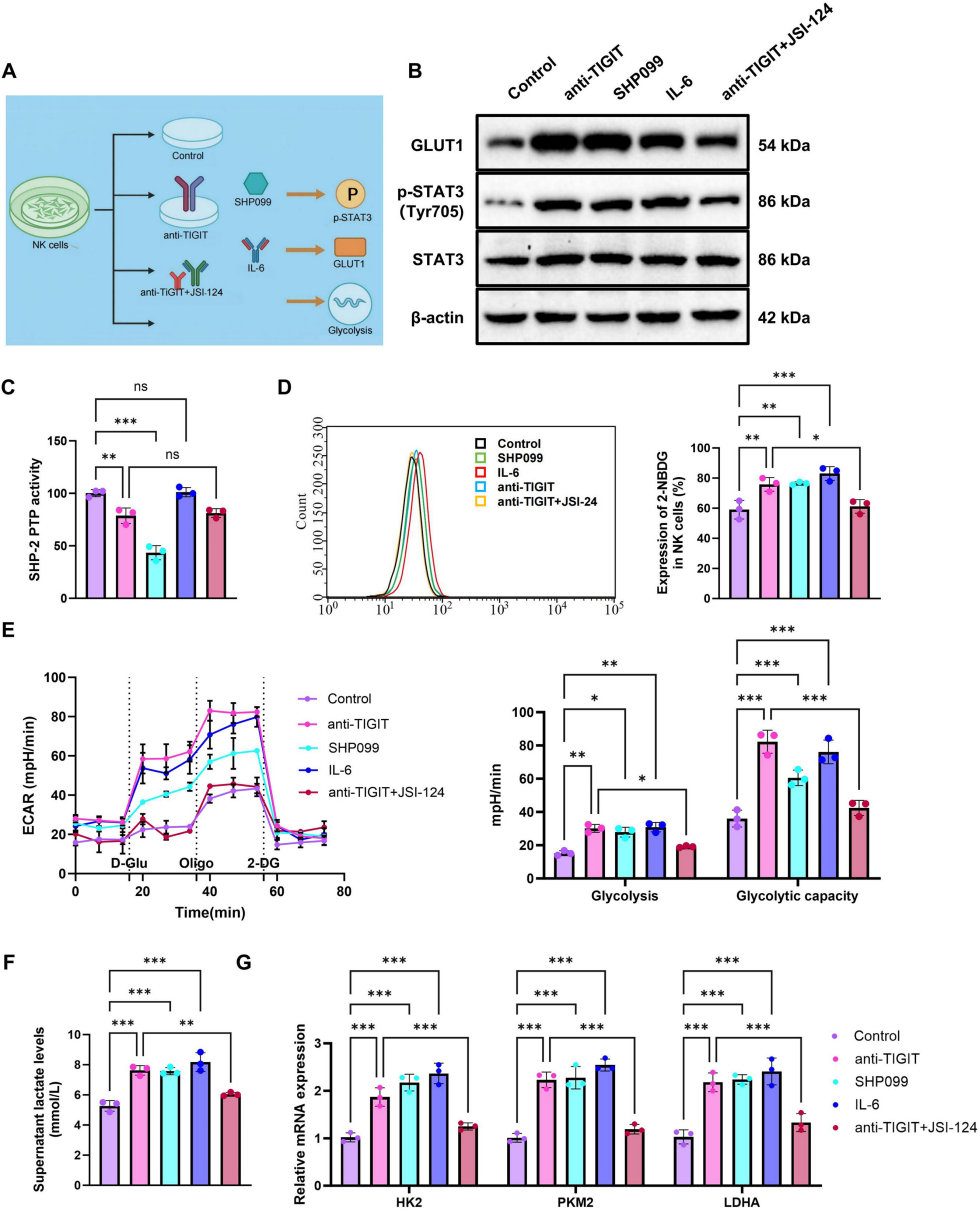
Modulation of GLUT1 expression and NK cell glycolytic activity through the TIGIT/SHP-2/STAT3 pathway. **(A)** Schematic illustration of the regulatory mechanism by which the TIGIT/SHP-2/STAT3 pathway influences GLUT1-mediated glycolysis; **(B)** WB analysis of GLUT1, phosphorylated STAT3 (Tyr705), and total STAT3 protein levels in NK cells; **(C)** Phosphatase activity assay to evaluate changes in SHP-2 enzyme activity in NK cells; **(D)** 2-NBDG fluorescence probe assay for assessing glucose uptake in NK cells; **(E)** Seahorse XF platform measurement of glycolytic flux (ECAR) in NK cells; **(F)** Lactate assay kit used to determine lactate concentration in the culture supernatant of NK cells; **(G)** RT-qPCR analysis of mRNA expression levels of HK2, PKM2, and LDHA in NK cells. Experiments were conducted in triplicate. *p < 0.05, **p < 0.01, ***p < 0.001.

To further examine whether the TIGIT signaling axis regulates GLUT1 expression and glycolytic metabolism in NK cells, five treatment groups were included: Control, anti-TIGIT, SHP099, IL-6, and anti-TIGIT plus JSI-124 (**Figure 4A**). SHP099 is an allosteric SHP-2 inhibitor that stabilizes the autoinhibited conformation of SHP-2 by binding to the tunnel-like pocket formed by the N-SH2, C-SH2, and PTP domains, thereby suppressing its phosphatase activity (33, 34). WB analysis showed that, relative to the Control group, the anti-TIGIT group displayed increased GLUT1 protein expression and STAT3 phosphorylation. Similar changes were also observed in the SHP099 and IL-6 groups. In contrast, compared with the anti-TIGIT group, the anti-TIGIT+JSI-124 group showed reduced GLUT1 expression and lower STAT3 phosphorylation (**Figure 4B**). Concurrently, the phosphatase activity of SHP-2 was assessed, indicating a significant decrease in activity in both the anti-TIGIT and SHP099-treated groups compared to the Control group, while the IL-6 group showed no notable change. Furthermore, no significant variation in SHP-2 phosphatase activity was observed between the anti-TIGIT and anti-TIGIT + JSI-124 groups (**Figure 4C**). These findings suggest that TIGIT regulates GLUT1 expression by inhibiting STAT3 phosphorylation via SHP-2 activity.

Metabolic assays further supported this pattern. Compared with the Control group, the anti-TIGIT, SHP099, and IL-6 groups all showed significantly higher 2-NBDG fluorescence intensity, increased ECAR, and elevated lactate release, consistent with enhanced glycolytic activity. By contrast, relative to the anti-TIGIT group, the anti-TIGIT+JSI-124 group exhibited reduced 2-NBDG uptake, lower ECAR values, and decreased lactate production (**Figures 4D–F**). Consistently, RT-qPCR analysis showed that the mRNA levels of the glycolysis-related genes HK2, PKM2, and LDHA were significantly increased in the anti-TIGIT, SHP099, and IL-6 groups compared with the Control group, whereas these transcripts were reduced in the anti-TIGIT+JSI-124 group relative to the anti-TIGIT group (**Figure 4G**).

These results further support an association between the TIGIT/SHP-2/STAT3 pathway, GLUT1 expression, and the glycolytic state of NK cells.

### Metabolic reprogramming via GLUT1 modulates molecular expression and cytotoxic activity of NK cells

**Figure 5:**
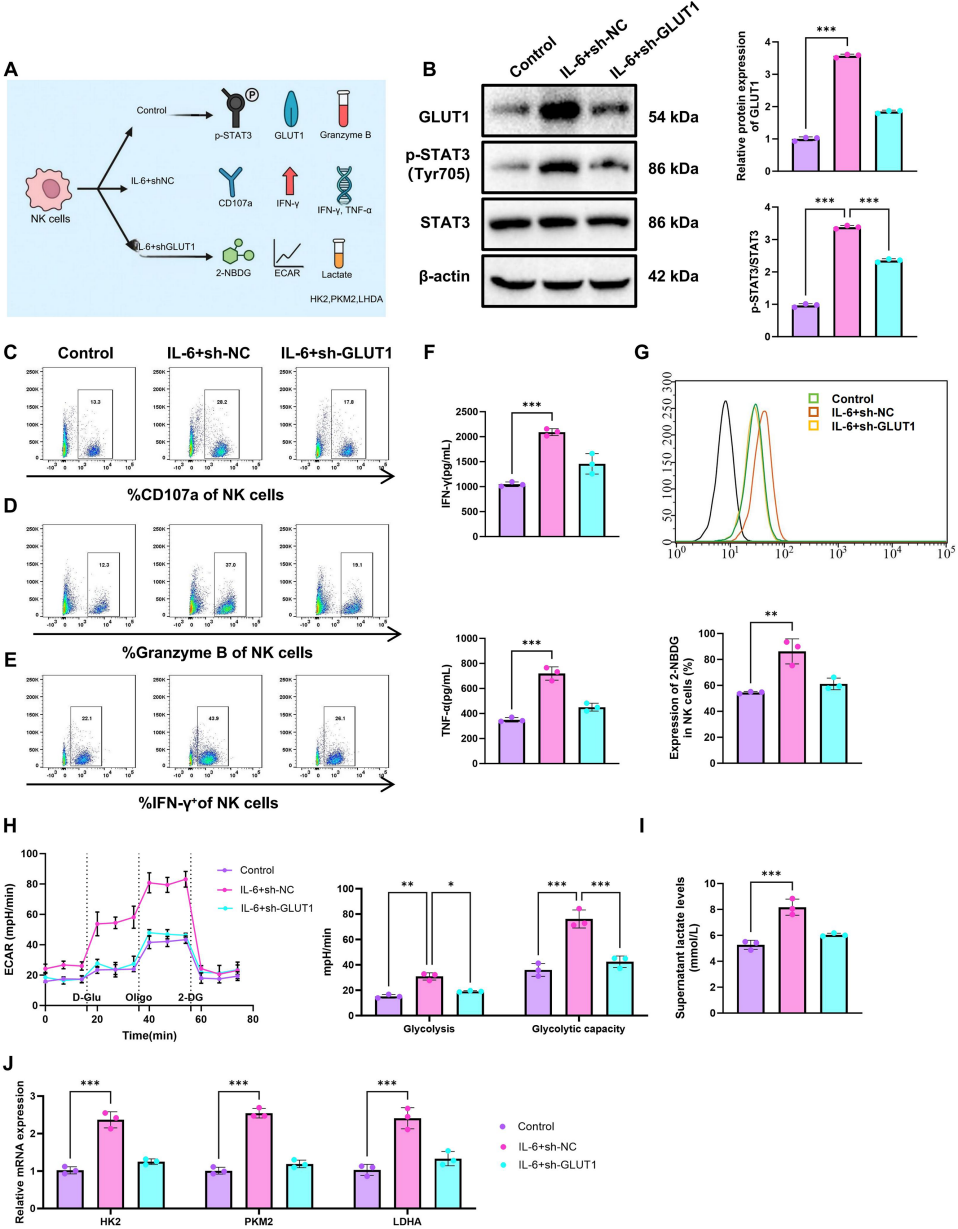
Metabolic reprogramming regulates NK cell function and cytotoxicity via GLUT1. **(A)** A schematic diagram illustrating the research approach where IL-6 is associated with metabolic reprogramming affecting NK cell function through GLUT1; **(B)** WB analysis of p-STAT3 and GLUT1 protein expression levels in NK cells; **(C–E)** flow cytometry analysis of CD107a expression **(C)**, IFN-γ expression **(D)**, and Granzyme B expression **(E)**; **(F)** ELISA measurement of IFN-γ and TNF-α secretion levels in the supernatant of cultured NK cells; **(G–I)** Assessment of glycolytic activity by measuring 2-NBDG fluorescence intensity **(G)**, ECAR values **(H)**, and lactate production levels **(I)**; **(J)** RT-qPCR analysis of HK2, PKM2, and LDHA mRNA expression levels. Experiments were conducted in triplicate. * indicates *p* < 0.05, **p < 0.01, ***p < 0.001.

To examine the contribution of glycolytic status to NK-cell function, three groups were included: Control, IL-6+sh-NC, and IL-6+sh-GLUT1 (**Figure 5A**). A lentiviral shRNA approach was first used to reduce GLUT1 expression in NK cells. RT-qPCR and WB analyses showed that both sh-GLUT1#1 and sh-GLUT1#2 reduced GLUT1 mRNA and protein expression relative to sh-NC, with sh-GLUT1#1 showing the stronger knockdown efficiency (**Supplementary Figures S3A, B**). Therefore, sh-GLUT1#1 was selected for subsequent GLUT1 knockdown experiments. WB analysis showed that, compared with the Control group, p-STAT3 and GLUT1 protein levels were increased in the IL-6+sh-NC group. In contrast, both proteins were reduced in the IL-6+sh-GLUT1 group relative to the IL-6+sh-NC group (**Figure 5B**). Flow cytometric analysis further showed that the percentages of CD107a-positive cells, as well as the expression of IFN-γ and granzyme B, were significantly increased in the IL-6+sh-NC group compared with the Control group, whereas all three indicators were significantly decreased in the IL-6+sh-GLUT1 group compared with the IL-6+sh-NC group (**Figures 5C–E**).

ELISA showed that IFN-γ and TNF-α secretion was significantly increased in the IL-6+sh-NC group relative to the Control group, but significantly reduced in the IL-6+sh-GLUT1 group relative to the IL-6+sh-NC group (**Figure 5F**). Metabolic assays showed that 2-NBDG uptake, ECAR, and lactate production were all significantly elevated in the IL-6+sh-NC group compared with the Control group, whereas these glycolytic indicators were significantly decreased after GLUT1 knockdown (**Figures 5G–I**). RT-qPCR analysis showed that the mRNA levels of HK2, PKM2, and LDHA were significantly increased in the IL-6+sh-NC group compared with the Control group, but were reduced in the IL-6+sh-GLUT1 group relative to the IL-6+sh-NC group (**Figure 5J**). Previous studies have shown that IL-6 can enhance glycolytic metabolism in NK cells (35). In the present study, IL-6 was used as a functional stimulus to examine the relationship between metabolic activity and NK-cell effector function. Under these conditions, GLUT1 knockdown attenuated the IL-6-associated increases in glycolytic activity and cytotoxic function, supporting a role for GLUT1-dependent metabolic reprogramming in this process. These findings are consistent with the involvement of the STAT3–GLUT1 axis as a downstream component of TIGIT-related metabolic regulation.

In summary, these results suggest that enhanced glycolytic metabolism is associated with increased NK-cell effector activity, whereas GLUT1 knockdown substantially weakens the metabolic and functional effects observed following IL-6 stimulation.

### Regulation of NK cell cytotoxicity against tumor cells via the TIGIT/STAT3/GLUT1 pathway

**Figure 6:**
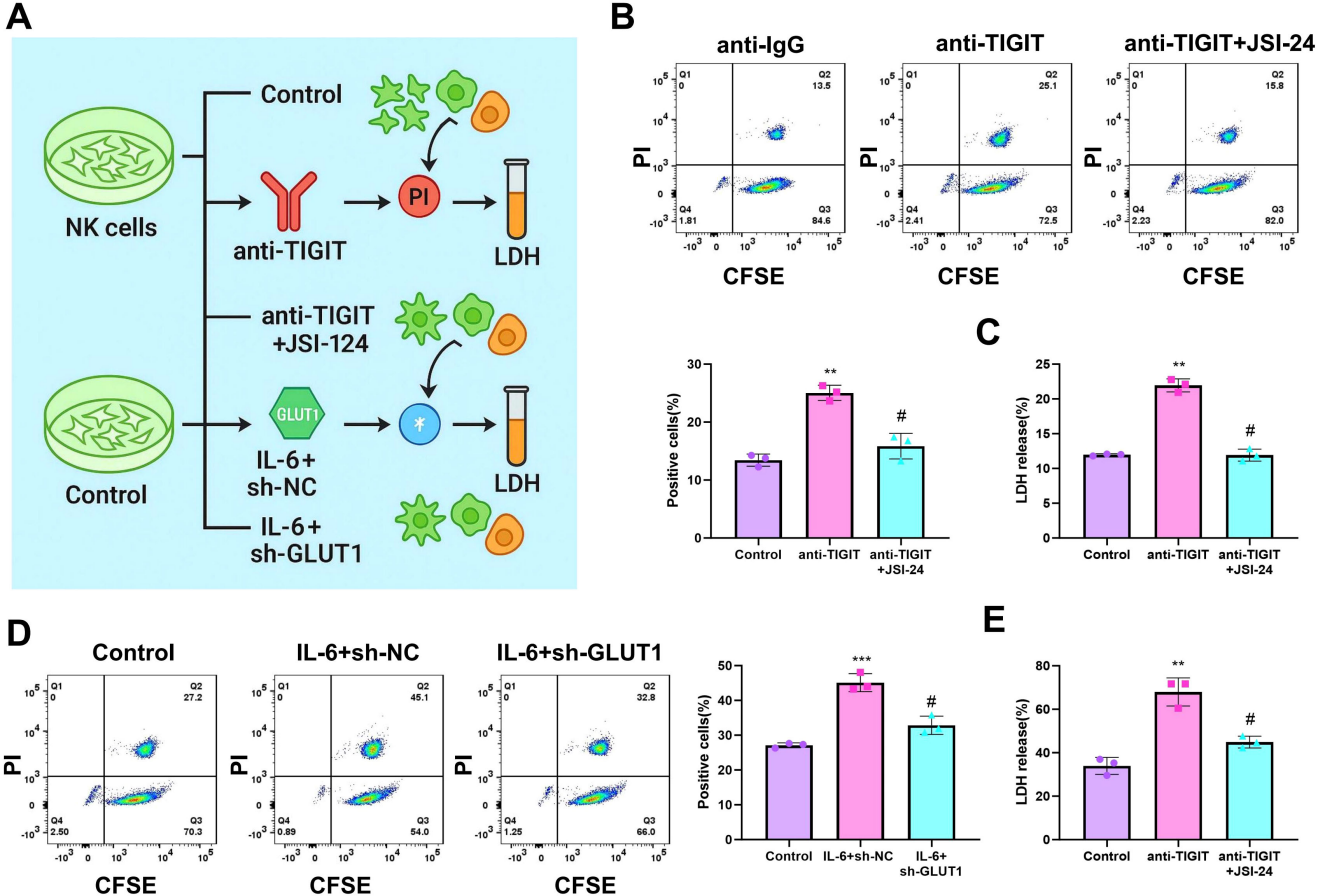
Regulation of NK cell cytotoxicity against tumor cells via the TIGIT/STAT3/GLUT1 pathway. **(A)** Schematic diagram illustrating the modulation of NK cell anti-tumor activity through interventions on the TIGIT-STAT3-GLUT1 axis; **(B, D)** flow cytometry combined with CFSE/PI staining to assess the death rate of Hepa1–6 hepatocarcinoma cells, evaluating NK cell cytotoxicity; **(C, E)** LDH release assay measuring cell lysis levels in co-culture systems. Experiments were repeated three times. ** indicates *p* < 0.01 compared to the Control group, ***p < 0.001; # indicates *p* < 0.05 compared to the anti-TIGIT or IL-6+sh-NC groups.

To determine whether metabolic reprogramming influences the tumoricidal activity of NK cells, two sets of experiments were performed: (1) Control, anti-TIGIT, and anti-TIGIT+JSI-124 groups; and (2) Control, IL-6+sh-NC, and IL-6+sh-GLUT1 groups. Treated NK cells were co-cultured with Hepa1–6 HCC cells, and their cytotoxic effects were subsequently evaluated (**Figure 6A**). Flow cytometric analysis showed that the proportion of PI-positive dead target cells was significantly higher in the anti-TIGIT group than in the Control group. In contrast, the proportion of dead target cells was significantly reduced in the anti-TIGIT+JSI-124 group relative to the anti-TIGIT group (**Figure 6B**). Consistently, LDH release assays showed that LDH release was markedly increased in the anti-TIGIT group compared with the Control group, whereas this increase was attenuated in the anti-TIGIT+JSI-124 group compared with the anti-TIGIT group (**Figure 6C**). These results indicate that blocking TIGIT enhances the cytotoxic effect of NK cells against tumor cells, whereas inhibition of the STAT3 signaling pathway can partially reverse this enhancement.

To further examine the relationship between metabolic activation and cytotoxic function, IL-6-treated NK cells were co-cultured with Hepa1–6 cells. Flow cytometry showed that the proportion of PI-positive target cells was significantly increased in the IL-6+sh-NC group compared with the Control group. Similarly, LDH release was also significantly elevated in the IL-6+sh-NC group, consistent with enhanced cytotoxic activity after metabolic activation. However, both PI positivity and LDH release were significantly reduced in the IL-6+sh-GLUT1 group compared with the IL-6+sh-NC group, indicating that GLUT1 knockdown attenuated the increase in NK-cell cytotoxicity associated with IL-6 stimulation (**Figures 6D, E**).

Together, these results support an association between the TIGIT/STAT3/GLUT1 axis, metabolic state, and the cytotoxic activity of NK cells against tumor cells.

### The TIGIT-CD155 axis modulates the SHP-2/STAT3/GLUT1 pathway influencing the *in vivo* antitumor activity of NK cells

**Figure 7:**
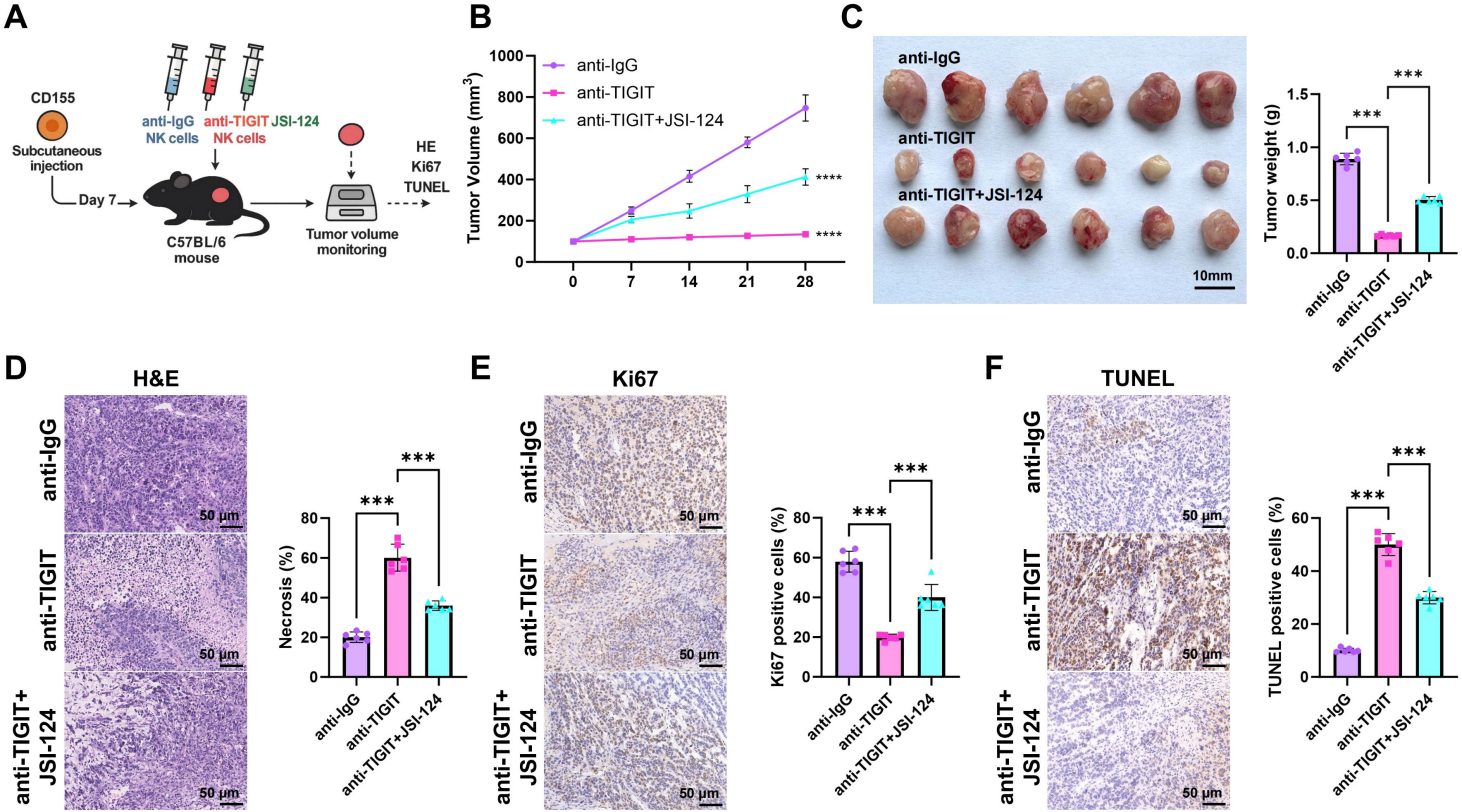
Regulation of the TIGIT-CD155 axis on the SHP-2/STAT3/GLUT1 pathway influencing NK cell anti-tumor activity *in vivo*. **(A)** Schematic representation of the murine subcutaneous liver cancer xenograft model; **(B)** Tumor volume changes over time measured by calipers for each group of mice; **(C)** Imaging and weight measurement of excised terminal tumor tissues; **(D)** H&E staining to observe necrotic regions in tumor tissues, scale bar: 50 μm; **(E)** IHC analysis for Ki67 expression levels in tumor tissues, scale bar: 50 μm; **(F)** TUNEL staining to assess apoptosis in tumor tissues, scale bar: 50 μm. Each group consists of 6 mice, with experiments repeated three times. * indicates comparison between groups, ***p < 0.001m, ****p < 0.0001.

To validate the regulatory role of the TIGIT-STAT3-GLUT1 signaling axis in NK cell antitumor activity, we established a CD155-overexpressing Hepa1–6 HCC cell line and generated a subcutaneous xenograft model in mice. Three intervention groups were included for comparison: Hepa1-6-CD155 + anti-IgG NK cells (anti-IgG), Hepa1-6-CD155 + anti-TIGIT NK cells (anti-TIGIT), and Hepa1-6-CD155 + anti-TIGIT + JSI-124 NK cells (anti-TIGIT+JSI-124) (**Figure 7A**). Dynamic monitoring of tumor growth showed that tumor volume was significantly lower in the anti-TIGIT group than in the anti-IgG group. In contrast, tumor growth was partially restored in the anti-TIGIT+JSI-124 group compared with the anti-TIGIT group (**Figure 7B**). At the end of the experiment, tumors were excised, photographed, and weighed. Final tumor weight was significantly reduced in the anti-TIGIT group relative to the anti-IgG group, whereas tumor weight was significantly increased in the anti-TIGIT+JSI-124 group compared with the anti-TIGIT group (**Figure 7C**). H&E staining results further showed a larger necrotic area in tumors from the anti-TIGIT group than in those from the anti-IgG group, whereas necrotic regions were reduced in the anti-TIGIT+JSI-124 group relative to the anti-TIGIT group (**Figure 7D**).

Immunohistochemical analysis showed that Ki67-positive staining was significantly reduced in the anti-TIGIT group compared with the anti-IgG group, whereas Ki67 expression increased in the anti-TIGIT+JSI-124 group relative to the anti-TIGIT group (**Figure 7E**). Consistently, TUNEL staining showed a significant increase in apoptotic cells in the anti-TIGIT group compared with the anti-IgG group, whereas the number of TUNEL-positive cells was reduced in the anti-TIGIT+JSI-124 group relative to the anti-TIGIT group (**Figure 7F**).

These *in vivo* results suggest that TIGIT blockade enhances the antitumor activity of NK cells, whereas inhibition of STAT3 attenuates this effect.

### Regulation of NK cell glycolysis and function via the TIGIT-CD155 axis through the SHP-2/STAT3/GLUT1 pathway

To further assess the effects of pathway intervention on NK-cell infiltration and functional status in tumor tissues, immunohistochemical staining was performed. Compared with the anti-IgG group, the anti-TIGIT group showed significantly increased expression of the NK-cell markers CD49b and NKp46, suggesting increased NK-cell infiltration. In contrast, expression of both markers was reduced in the anti-TIGIT+JSI-124 group relative to the anti-TIGIT group (**Supplementary Figures S4A, B**). Concurrently, flow cytometric analysis showed that IFN-γ and granzyme B expression was significantly increased in the anti-TIGIT group compared with the anti-IgG group, whereas both markers were reduced in the anti-TIGIT+JSI-124 group relative to the anti-TIGIT group (**Supplementary Figures S4C, D**).

Immunofluorescence staining was further used to examine the expression of key signaling molecules together with their localization relative to NK-cell infiltration. The expression intensities of GLUT1 and p-STAT3 were significantly higher in tumor tissues from the anti-TIGIT group than in those from the anti-IgG group, and these signals were mainly localized to NKp46-positive regions. By contrast, both GLUT1 and p-STAT3 signals were reduced in the anti-TIGIT+JSI-124 group compared with the anti-TIGIT group (**Supplementary Figure S4E**).

These results suggest that TIGIT blockade is associated with enhanced NK-cell infiltration, increased effector molecule expression, and elevated glycolysis-related signaling *in vivo*, whereas STAT3 inhibition weakens these changes.

## Discussion

This study investigated how the TIGIT/CD155 axis is associated with immune evasion in the HCC microenvironment through regulation of glycolytic metabolism in NK cells. We found that NK-cell subsets with high TIGIT expression were enriched in HCC tissues and displayed features consistent with functional impairment, including reduced expression of cytotoxic molecules, decreased degranulation capacity, lower STAT3 phosphorylation, and reduced expression of the downstream metabolic regulator GLUT1, together suggesting a metabolically constrained state. Further analyses indicated that tumor-derived CD155 engaged TIGIT on NK cells and was associated with increased recruitment of SHP-2 and enhanced SHP-2–STAT3 interaction. This was accompanied by reduced phosphorylation of STAT3 at Tyr705, lower GLUT1 expression, and diminished glycolytic activity, which may contribute to impaired antitumor function of NK cells.

Our findings suggest a previously underexplored link between TIGIT signaling and glycolytic regulation in NK cells through the SHP-2/STAT3 axis. Previous studies have largely characterized TIGIT as a classical inhibitory immune checkpoint on the basis of its suppressive effects on immune-cell effector function, including reduced cytokine production and cytotoxic molecule expression (20, 36, 37). By contrast, our data indicate that TIGIT is associated with increased SHP-2 recruitment, reduced STAT3 phosphorylation, lower GLUT1 expression, and restricted glycolytic activity in NK cells. In addition, GLUT1 knockdown attenuated the functional enhancement observed after STAT3 activation, supporting a close relationship between metabolic reprogramming and TIGIT-associated immunosuppression. Together, these findings extend the current understanding of TIGIT beyond its established role as an inhibitory immune checkpoint and suggest that it may also contribute to the regulation of metabolic fitness in NK cells, with potential implications for combination strategies involving metabolic intervention.

Within the TME, CD155 is a major ligand for TIGIT and an important initiator of this signaling pathway (18, 38). Through integrated multi-omics analyses, we found that CD155 was predominantly derived from tumor cells and was markedly upregulated in tumor tissues. In ligand–receptor interaction analysis, CD155 emerged as a prominent component of NK cell–tumor cell crosstalk. Our data further suggest that CD155–TIGIT engagement is associated with increased TIGIT-related inhibitory signaling and enhanced SHP-2 activity. Previous studies have shown that SHP-2, a broadly expressed tyrosine phosphatase, can regulate immune-cell signaling through dephosphorylation of STAT family proteins (19). Consistent with this, our results support an association between high TIGIT expression, increased SHP-2–STAT3 interaction, and reduced STAT3 phosphorylation, which may in turn influence downstream metabolic regulation.

The STAT3–GLUT1 pathway is considered an important regulator of glucose metabolism in NK cells (39). Upon activation, STAT3 phosphorylation can promote GLUT1 expression, thereby facilitating glucose uptake and glycolytic flux to support granule release and cytokine production (26). In the present study, TIGIT-associated SHP-2 activation was accompanied by reduced p-STAT3 levels, decreased GLUT1 expression, and suppressed glycolytic activity, as reflected by lower ECAR, reduced 2-NBDG uptake, and decreased lactate production. In addition, GLUT1 knockdown attenuated the metabolic and functional changes induced by STAT3 activation, supporting the view that GLUT1 is an important downstream effector in TIGIT-associated metabolic regulation.

Our *in vivo* findings further support the relevance of this pathway to NK-cell antitumor activity. TIGIT blockade was associated with reduced tumor growth, increased NK-cell infiltration, and higher expression of functional molecules, whereas inhibition of STAT3 partially weakened these effects. Histopathological analyses, including TUNEL, Ki67, CD49b, and NKp46 staining, together with flow cytometry results, were consistent with a role for the TIGIT/SHP-2/STAT3/GLUT1 axis in the maintenance of NK-cell functional activity *in vivo*. These observations suggest that this pathway may provide a useful framework for developing combined strategies targeting both immune checkpoints and metabolic regulation.

An additional strength of this study lies in the integration of multiple levels of evidence. By combining single-cell transcriptomic analysis, functional validation experiments, and *in vivo* models, we identified a signaling framework linking TIGIT/CD155 engagement to intracellular metabolic regulation in NK cells through the SHP-2/STAT3/GLUT1 axis. In contrast to other immune checkpoints, such as PD-1 or LAG-3, which have more often been associated with PI3K–Akt-related metabolic pathways, our data suggest that TIGIT-mediated metabolic regulation in NK cells may involve a greater contribution from the SHP-2–STAT3 axis. This distinction may be relevant for understanding the signaling characteristics of TIGIT and for guiding future therapeutic investigation.

From a translational perspective, anti-TIGIT antibodies such as tiragolumab have entered combination immunotherapy trials for solid tumors, although their activity as monotherapy has remained limited (40, 41). One possible explanation is that reversal of checkpoint inhibition alone may be insufficient when immune cells remain metabolically constrained. In this context, the STAT3–GLUT1 axis identified in the present study may represent an additional point for therapeutic intervention beyond TIGIT blockade itself. Combined strategies incorporating checkpoint inhibition together with metabolic support may therefore warrant further investigation as a means to improve NK-cell functional restoration within the TME.

Several limitations of this study should be acknowledged. First, the sample size for single-cell RNA sequencing was limited, with one sample per group. Accordingly, these analyses were used primarily to characterize immune-cell subsets and identify candidate signaling pathways, and the findings will require validation in larger single-cell cohorts. In addition, because the immune microenvironments of normal liver tissue and subcutaneous tumor tissue differ substantially, this comparison was intended mainly to describe immune composition and identify candidate pathways rather than to directly infer changes in immune-cell abundance. Moreover, because TIGIT-positive NK cells were relatively few in number, the single-cell differential expression analysis should be interpreted primarily as trend-level evidence. Second, although the *in vivo* experiments showed that TIGIT blockade was associated with restoration of both NK-cell glycolytic activity and antitumor function, metabolic activation and functional activation are intrinsically linked and difficult to separate completely. Therefore, the causal relationship between metabolic reprogramming and antitumor activity still requires further clarification through temporal analyses or more direct metabolic perturbation strategies. Third, the present study was based mainly on mouse models and primary murine NK cells. Although the findings were supported consistently across multiple experiments, whether the same mechanism is conserved in human HCC tissues or in peripheral blood mononuclear cell-derived NK cells remains to be determined. In view of the heterogeneity of NK-cell populations, it will also be important to investigate whether distinct subsets, such as memory-like or adaptive NK cells, respond differently to this pathway. In addition, although multi-node interventions, including TIGIT blockade, SHP-2 inhibition, STAT3 activation, and GLUT1 knockdown, supported the involvement of the CD155–TIGIT/SHP-2/STAT3/GLUT1 pathway in metabolic regulation, these approaches do not constitute direct structural validation. Future studies using motif-specific or domain-specific point mutants will be needed to determine whether disruption of these signaling elements is sufficient to abolish the observed effects. Finally, although our data support an association between the TIGIT/CD155 axis and glycolytic reprogramming in NK cells, we did not systematically assess additional metabolic regulators such as HIF-1α, which has also been implicated in glycolytic control (42). Future studies incorporating hypoxia-mimicking conditions, HIF-1α inhibition, or HIF-1α overexpression may help clarify its contribution to TIGIT-associated metabolic regulation and provide a more complete view of NK-cell immunometabolism.

In summary, the present study suggests that the TIGIT/CD155 axis is associated with impaired glycolytic metabolism in NK cells through SHP-2-dependent suppression of STAT3 phosphorylation and reduced GLUT1 expression. This metabolic constraint was accompanied by weakened NK-cell effector function and may contribute to immune evasion in HCC. These findings provide a framework linking immune checkpoint signaling to metabolic regulation in NK cells and may inform the development of combination strategies aimed at restoring antitumor immunity through both checkpoint blockade and metabolic intervention.

## Data Availability

The original contributions presented in the study are included in the article/**Supplementary Material**. Further inquiries can be directed to the corresponding authors.

## Supplementary material

Supplementary Figure S1 Identification of Immune Cell Composition in Normal and HCC Tissues by scRNA-seq. **(A)** UMAP visualization and clustering of immune cells from normal and hepatocellular carcinoma tissues based on scRNA-seq; **(B)** DotPlot showing the expression levels and positive fractions of marker genes across immune cell populations; **(C)** Bar plot comparing the proportions of immune cell populations between normal and tumor tissues. Normal group: n = 1; Tumor group: n = 1.

Supplementary Figure S2 Differential Gene Analysis and Network Construction Identify TIGIT-associated Targets. **(A)** Volcano plot of differentially expressed genes between normal and hepatocellular carcinoma tissues based on bulk RNA-seq; **(B)** LASSO regression analysis identifying feature genes among differentially expressed candidates; **(C)** Gene interaction network highlighting key target genes and their interactions. Normal group: n = 4; Tumor group: n = 4.

Supplementary Figure S3 Validation of GLUT1 Silencing Efficiency in NK Cells via Lentiviral Knockdown. **(A)** RT-qPCR analysis of GLUT1 mRNA expression levels in NK cells across the sh-NC, sh-GLUT1#1, and sh-GLUT1#2 groups; **(B)** WB analysis of GLUT1 protein expression levels in the groups above. The cell experiments were conducted in triplicate. * indicates comparison between groups, ****p* < 0.001.

Supplementary Figure S4 Verification of the Role of the TIGIT-CD155 Axis in Regulating the SHP-2/STAT3/GLUT1 Pathway and Its Impact on NK Cell Glycolysis and Function in Tumor Tissues. **(A, B)** IHC was employed to assess the expression of NK cell markers CD49b and NKp46 within tumor tissues, evaluating the density of NK cell infiltration, bar: 50 μm; **(C, D)** flow cytometry was utilized to analyze the levels of IFN-γ and Granzyme B expressed by infiltrating NK cells in tumor tissues, reflecting their effector functions; **(E)** Immunofluorescence staining was conducted to detect GLUT1 and p-STAT3 expressions in tumor tissues, combined with colocalization analysis using the NKp46 marker, bar: 10 μm. Each group consisted of n=6 mice. * indicates a comparison between groups, **p* < 0.05, ***p* < 0.01, ****p* < 0.001.

## Glossary

CD155: Cluster of Differentiation 155
CFSE: Carboxyfluorescein Succinimidyl Ester
Co-IP: Co-immunoprecipitation
DEGs: Differentially Expressed Genes
ECAR: Extracellular Acidification Rate
ELISA: Enzyme-linked Immunosorbent Assay
FACS: Fluorescence-Activated Cell Sorting
gMFI: Geometric Mean Fluorescence Intensity
GLUT1: Glucose Transporter Type 1
GSEA: Gene Set Enrichment Analysis
H&E: Hematoxylin & Eosin
HCC: Hepatocellular Carcinoma
HG: High Glucose
ICIs: Immune Checkpoint Inhibitors
IFN-γ: Interferon-gamma
IHC: Immunohistochemistry
ITIM: Immunoreceptor Tyrosine-based Inhibitory Motif
ITSM: Immunoreceptor Tyrosine-based Switch Motif
KEGG: Kyoto Encyclopedia of Genes and Genomes
LDH: Lactate Dehydrogenase
mTOR: mechanistic Target of Rapamycin
NK: Natural Killer
PCA: Principal Component Analysis
PI: Propidium Iodide
RT-qPCR: reverse transcription quantitative PCR
scRNA-seq: Single-cell RNA Sequencing
SHP-2: Src Homology 2 Domain-containing Phosphatase 2
STAT3: Signal Transducer and Activator of Transcription 3
TGI: Tumor Growth Inhibition Rate
TIGIT: T cell immunoreceptor with Ig and ITIM domains
TILs: Tumor-infiltrating Lymphocytes
UMAP: Uniform Manifold Approximation and Projection
WB: Western Blot
