# Supplementary figures and images for: Targeting the TIGIT/CD155-induced metabolic checkpoint in NK cells restores anti-tumor immunity and suppresses hepatocellular carcinoma growth
Source: Front Immunol. 2026 May 1;17:1790174. doi: 10.3389/fimmu.2026.1790174 (PMC13176292; doi:10.3389/fimmu.2026.1790174)

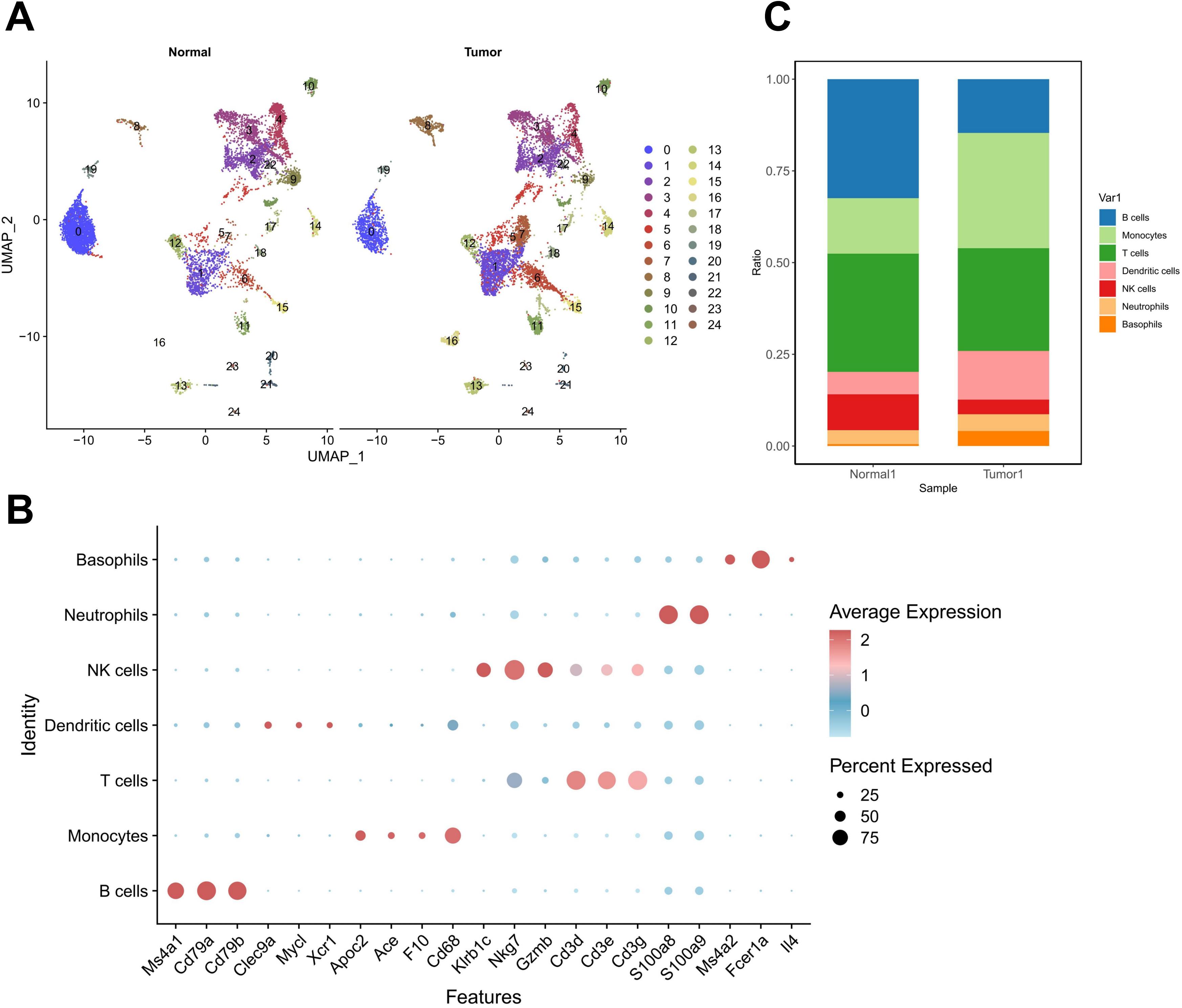

Supplement: Supplementary Figure S1 — Identification of Immune Cell Composition in Normal and HCC Tissues by scRNA-seq. (A) UMAP visualization and clustering of immune cells from normal and hepatocellular carcinoma tissues based on scRNA-seq; (B) DotPlot showing the expression levels and positive fractions of marker genes across immune cell populations; (C) Bar plot comparing the proportions of immune cell populations between normal and tumor tissues. Normal group: n = 1; Tumor group: n = 1. [file Image1.jpeg]

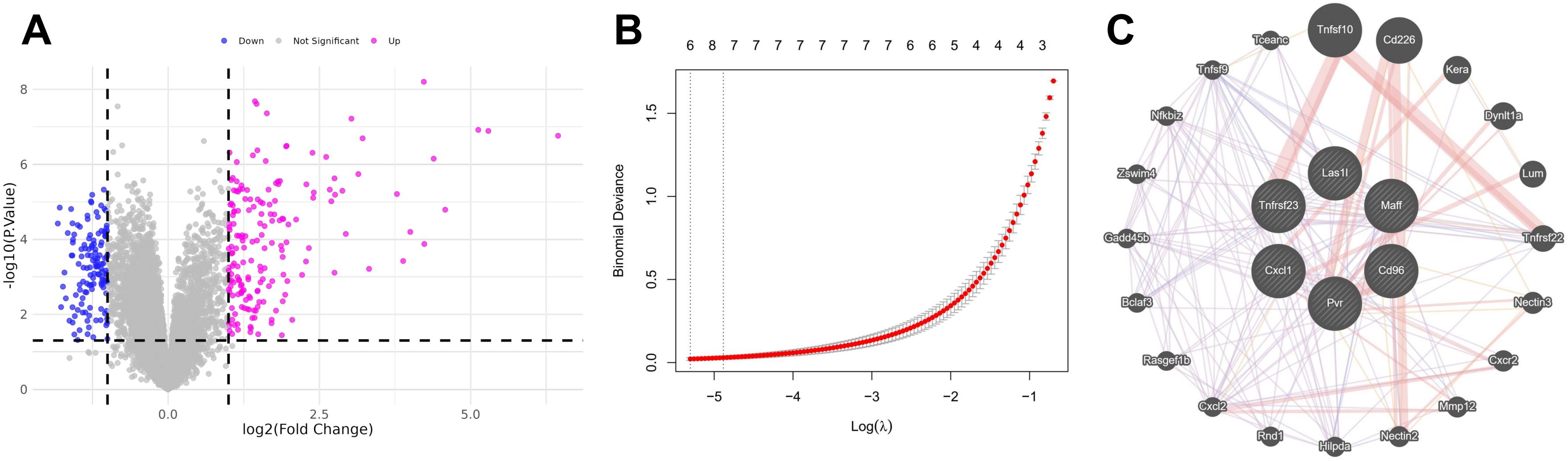

Supplement: Supplementary Figure S2 — Differential Gene Analysis and Network Construction Identify TIGIT-associated Targets. (A) Volcano plot of differentially expressed genes between normal and hepatocellular carcinoma tissues based on bulk RNA-seq; (B) LASSO regression analysis identifying feature genes among differentially expressed candidates; (C) Gene interaction network highlighting key target genes and their interactions. Normal group: n = 4; Tumor group: n = 4. [file Image2.jpeg]

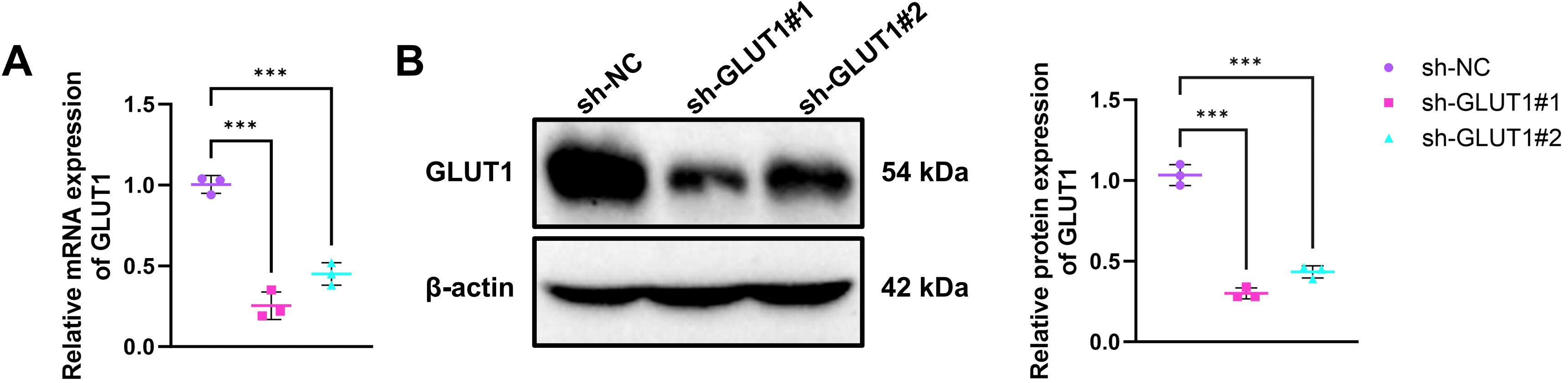

Supplement: Supplementary Figure S3 — Validation of GLUT1 Silencing Efficiency in NK Cells via Lentiviral Knockdown. (A) RT-qPCR analysis of GLUT1 mRNA expression levels in NK cells across the sh-NC, sh-GLUT1#1, and sh-GLUT1#2 groups; (B) WB analysis of GLUT1 protein expression levels in the groups above. The cell experiments were conducted in triplicate. * indicates comparison between groups, ***p < 0.001. [file Image3.jpeg]

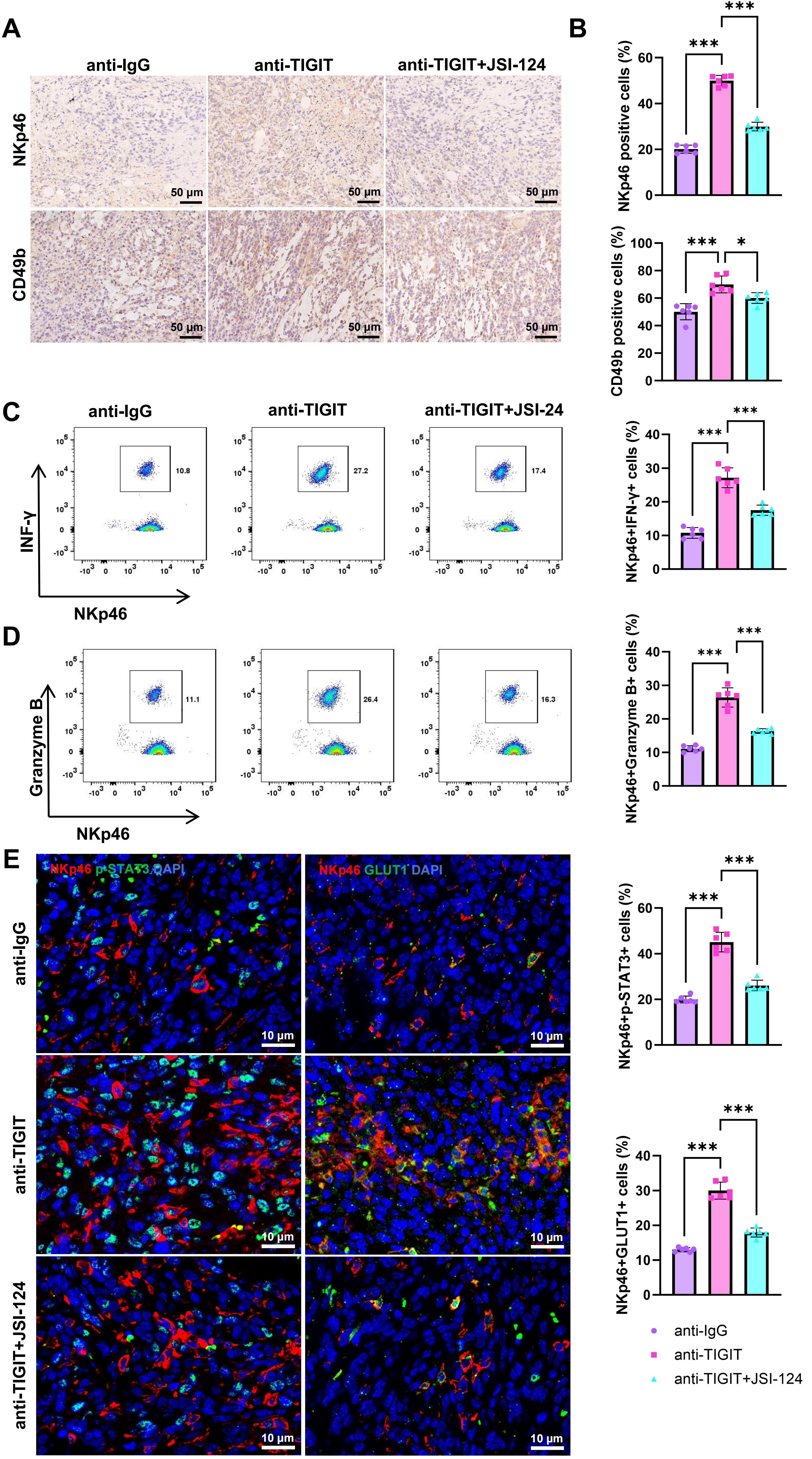

Supplement: Supplementary Figure S4 — Verification of the Role of the TIGIT-CD155 Axis in Regulating the SHP-2/STAT3/GLUT1 Pathway and Its Impact on NK Cell Glycolysis and Function in Tumor Tissues. (A, B) IHC was employed to assess the expression of NK cell markers CD49b and NKp46 within tumor tissues, evaluating the density of NK cell infiltration, bar: 50 μm; (C, D) flow cytometry was utilized to analyze the levels of IFN-γ and Granzyme B expressed by infiltrating NK cells in tumor tissues, reflecting their effector functions; (E) Immunofluorescence staining was conducted to detect GLUT1 and p-STAT3 expressions in tumor tissues, combined with colocalization analysis using the NKp46 marker, bar: 10 μm. Each group consisted of n=6 mice. * indicates a comparison between groups, *p < 0.05, **p < 0.01, ***p < 0.001. [file Image4.jpeg]
