# Supplementary material for: Targeting the TIGIT/CD155-induced metabolic checkpoint in NK cells restores anti-tumor immunity and suppresses hepatocellular carcinoma growth
Source: Front Immunol. 2026 May 1;17:1790174. doi: 10.3389/fimmu.2026.1790174 (PMC13176292; doi:10.3389/fimmu.2026.1790174)
Supplement: Supplementary file 5 [file Table1.docx]

**Table S1. RT-qPCR** **primer sequence (mouse)**

| **Gene ID** | **primer sequence (5’-3’)** |
| --- | --- |
| LDHA-F | AACTTGGCGCTCTACTTGCT |
| LDHA-R | TAGCCGCCTGAGGACTTACT |
| PKM2-F | GCTCTAGGTATCGCAGCAGG |
| PKM2-R | GTAAGCGTTGTCCAGGGTGA |
| GLUT1-F | GGCTTCTCCAACTGGACCTC |
| GLUT1-R | CCGGAAGCGATCTCATCGAA |
| HK2-F | GAAGAAGCTCCCGCTGGGTT |
| HK2-R | CCACATCTCTGCCTTCCACG |
| β-actin-F | CAGCTTCTTTGCAGCTCCTT |
| β-actin-R | AGAAGCACTTGCGGTGCAC |

Note: F: Forward; R: Reverse.
